# Acute and chronic hypoxia differentially predispose lungs for metastases

**DOI:** 10.1038/s41598-019-46763-y

**Published:** 2019-07-15

**Authors:** Moritz Reiterer, Renato Colaço, Pardis Emrouznejad, Anders Jensen, Helene Rundqvist, Randall S. Johnson, Cristina Branco

**Affiliations:** 1 Queen’s University Belfast, Centre for Cancer Research and Cell Biology, Belfast, UK; 2 Department of Physiology, Development and Neuroscience, University of Cambridge, Cambridge, UK; 3 Karolinska Institutet, Stockholm, Sweden

**Keywords:** Metastasis, Metastasis

## Abstract

Oscillations in oxygen levels affect malignant cell growth, survival, and metastasis, but also somatic cell behaviour. In this work, we studied the effect of the differential expression of the two primary hypoxia inducible transcription factor isoforms, HIF-1α and HIF-2α, and pulmonary hypoxia to investigate how the hypoxia response of the vascular endothelium remodels the lung pre-metastatic niche. Molecular responses to acute versus chronic tissue hypoxia have been proposed to involve dynamic HIF stabilization, but the downstream consequences and the extent to which differential lengths of exposure to hypoxia can affect HIF-isoform activation and secondary organ pre-disposition for metastasis is unknown. We used primary pulmonary endothelial cells and mouse models with pulmonary endothelium-specific deletion of HIF-1α or HIF-2α, to characterise their roles in vascular integrity, inflammation and metastatic take after acute and chronic hypoxia. We found that acute hypoxic response results in increased lung metastatic tumours, caused by HIF-1α-dependent endothelial cell death and increased microvascular permeability, in turn facilitating extravasation. This is potentiated by the recruitment and retention of specific myeloid cells that further support a pro-metastatic environment. We also found that chronic hypoxia delays tumour growth to levels similar to those seen in normoxia, and in a HIF-2α-specific fashion, correlating with increased endothelial cell viability and vascular integrity. Deletion of endothelial HIF-2α rendered the lung environment more vulnerable to tumour cell seeding and growth. These results demonstrate that the nature of the hypoxic challenge strongly influences the nature of the endothelial cell response, and affects critical parameters of the pulmonary microenvironment, significantly impacting metastatic burden. Additionally, this work establishes endothelial cells as important players in lung remodelling and metastatic progression.

## Introduction

Extravasation efficiency is a critical rate-limiting step of the metastatic cascade^1^ and vascular endothelial cells (EC) constitute the main barrier, affecting both blood flow and cell penetration to surrounding tissues^1–3^. Microvessels are typically the site of arrest of circulating tumour cells (CTC), often present from the onset of cancer, and these micro-occlusions impair tissue perfusion and reduce oxygen availability - transiently or long term - leading to altered EC behaviour and microvascular integrity^4,5^.

The hypoxia-sensitive family of Hypoxia Inducible factors (HIF) are key regulators of EC physiology and of the metabolic shifts underlying their responses to oxygenation and organ demand^6–9^. Endothelial HIF can be stabilized as a result of transient ischemic events^10^, such as the arrest of CTC in small capillaries^11^ but also in response to cytokine and tumour-derived signals, and indirectly via pharmacological agents or radiotherapy^12–14^.

Our previous work has shown that deletion of endothelial HIF-1α inhibits growth of lung metastasis in spontaneous breast cancer, implanted mammary gland tumours, or models of experimental metastasis, whereas deletion of HIF-2α increases metastatic frequency in all of these models^15,16^. The lungs are frequent hosts of metastatic tumours, and are often the first microvascular network encountered by CTC; additionally, the pulmonary vasculature is particularly responsive to changes in oxygenation. Most pulmonary hypoxic challenges are intermittent^17^, and can result from a myriad of pathologies or environmental challenges^18^, ranging from hypertension^19,20^, smoke exposure, cardiovascular and hepatic disease^21^, to sleep apnoea^22–24^. Importantly, it has been shown that intermittent hypoxia can lead to the stabilization of HIF-1α and decreases in HIF-2α levels in lung tissue^25,26^, with detrimental effects on cancer progression^17,22^. A number of groups have shown that each HIF isoform has downstream physiological consequences that are cell-type specific^22,27,28^, and the stabilization of either in different contexts can determine disease outcome.

We hypothesised that the stabilization of either of these HIF factors will have a role in modulating the lung microenvironment and affect its vulnerability to metastatic colonization depending on the relative abundance of HIF-1α and HIF-2α.

In this work, one or the other isoform of HIF transcription factors was selectively induced in the lung microenvironment using acute (for highest HIF-1α stabilization) and chronic (for highest HIF-2α stabilization) environmental hypoxia exposure. The efficiency of lung tumour seeding in the different pre-conditioning settings was characterized. Additionally, animals with lung endothelium-specific^29^ deletion of HIF isoforms were used to investigate which components of organ remodelling were mediated by EC-derived signals, and to establish the role of endothelial HIF in lung vulnerability to metastatic colonization.

The endothelial response to hypoxia is shown to remodel the lung microenvironment in a time-dependent manner: acute environmental hypoxia, driving stabilization of pulmonary HIF-1α results in a pro-metastatic environment, while chronic environmental hypoxia, and concomitantly higher levels of HIF-2α, reduces endothelium-mediated metastatic cell penetrance.

## Results

### EC response to hypoxia results in staggered and dynamic activation of HIF transcription factors

**Figure 1:**
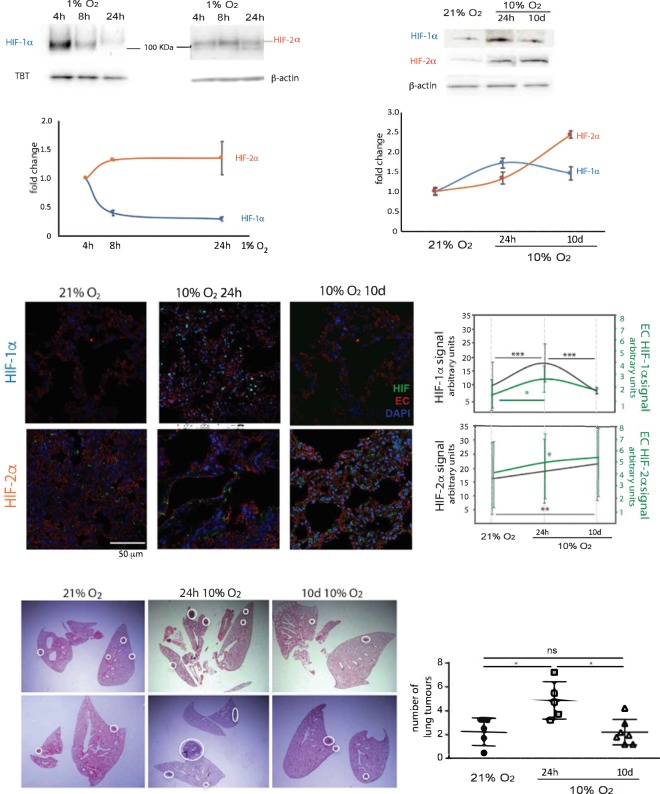
Distinct temporal patterns of activation of HIF-1 and HIF-2 primary lung endothelial cells and whole lung tissue, and impact on metastatic seeding. (**A**) Representative western blot of HIF-1α, HIF-2α and loading control (TBP- tata binding protein, or β-actin), from nuclear extracts of primary mouse lung EC exposed to a hypoxic (1% O_2_) or normal atmosphere (21% O_2_) over time. Graph below shows quantification of signal intensity of HIF signal for each time point, normalised to respective loading control; The signal of normoxic samples used for quantification, as well as additional replicates and whole membrane controls are presented in Supplementary Figs 7–9. (**B**) Whole lung HIF-1α and HIF-2α western blot (quantification underneath), from mice in normal atmosphere or housed in hypoxic chambers (10% O_2_) for a short exposure (24 h) or prolonged exposure (10d). HIF signal was normalized to β-actin. Data are Avg. fold change (hypoxia/normoxia) ± SD, n ≥ 3 per treatment; (**C**) Immunofluorescent detection of HIF-1α (top) and HIF-2α (bottom) signal in frozen lungs of mice maintained at 21% O_2_ (normoxia), or exposed to oxygen-deprived atmosphere (10% O_2_, hypoxia) for 24 h or 10d. Charts on the right represent the Avg. signal ± SD, obtained from a minimum of 10 evenly spaced lung sections from ≥ 5 mice per treatment, 5 images acquired per section; left y-axis represents total HIF intensity (grey line on the chart); endothelial HIF signal was calculated from co-localized signal between HIF and podocalyxin, and is shown on the right y-axis (green line on the chart). Significance assessed by student’s *t*-tests; *p < 0.05, ***p < 0.001 refers to comparisons between time-points; (**D**) Average number of lung tumours ± SD counted from a minimum of 20 evenly spaced paraffin sections of lungs harvested 14d post tumour cell injection, n ≥ 5 animals per group. Significance was assessed by students *t*-test with Welch’s correction, *p < 0.05. Illustrative H&E sections shown below, white circles highlight tumour lesions.

Primary lung EC were transferred to 1% O_2_ and HIF-α isoform activation was assessed over time. HIF-1α activation is seen at the shorter exposure time-points, peaking at 4 h and starting to decline after 8 h, when increased stabilization of HIF-2α becomes more evident (Fig. 1A), consistent with what has recently been reported for microvascular cells of human lung and other organs^30^. To investigate whether this pattern of HIF isoform expression is also seen *in vivo*, animals were exposed to environmental hypoxia (10% O_2_). After 24 h and 10d exposures, HIF activation was assessed in whole lung tissue by western blotting (Fig. 1B), and levels of both isoforms are increased in hypoxic lungs, irrespective of exposure length; however HIF-1α is preferentially activated after 24 h of hypoxia, and hardly detectable in lungs of mice exposed to normoxia or prolonged hypoxia; HIF-2α protein is seen in all time points, but highest after prolonged (10d) exposure to hypoxia (Fig. 1B). Total lung HIF signal was also investigated by immunofluorescence (Fig. 1C), and frozen tissue was stained for both HIF (green signal) and endothelial cells (Podocalyxin, red). In the panels to the right, average total HIF signal is shown for each isoform (grey line, left y-axis), as well as endothelial-derived HIF, quantified by co-localization of podocalyxin and HIF signal (green line, right y-axis). Even though HIF is expected to be stabilised in multiple cell types in response to hypoxia, the activation pattern of endothelial HIF follows that seen in whole lung tissue, and confirms the trend seen in Fig. 1B.

The time-points and activation patterns seen *in vivo* do not fully overlap with the ones seen *ex-vivo*. This is likely a result of the fact that atmospheric O_2_ levels used to trigger a hypoxic response in the lung endothelium are different from those necessary to trigger the same response in EC in the tissue culture environment. The *in-vivo* model was used to reflect the effect of endothelial HIF in modulating metastatic success, independently of tumour-derived signals. Thus, acute and chronic hypoxia were defined as the treatments yielding the highest activation of HIF-1α or HIF-2α, respectively, and these conditions were subsequently used to test differential organ susceptibility to metastatic tumour colonization, as a function of EC activation status.

### Hypoxia pre-conditioning affects metastatic tumour burden in a time-dependent manner

To assess the effect on organ susceptibility to colonization by CTC after hypoxia, animals in each pre-treated group received tumour cells intravenously, as illustrated in Supplementary Fig. 1A,B. Lung tumours were counted by H&E staining of paraffin-embedded sections obtained 14d post injection, during which animals were maintained in normal room air (Supplementary Fig. 1B,C).

Mice exposed to acute hypoxia, showing the highest lung HIF-1α levels (Fig. 1B,C), had more tumours than the controls breathing room air, whereas mice exposed to chronic hypoxia developed lung tumours in numbers similar to those found in normoxic controls (Fig. 1D).

### Microvascular permeability transiently increases during adaptation to hypoxia

Lung microvascular permeability was quantified using intravenous administration of Evans Blue (EB) to investigate if higher tumour incidence is the result of improved extravasation efficiency, due to increased vascular leakage. EB leaked from the lung microvasculature was quantified by spectrometry of the bronchoalveolar lavage of a minimum of 5 animals per group, collected 20 min post EB injection. An increase in permeability was seen after 24 h of hypoxia (Supplementary Fig. 2A) and correlates with increased number of lung tumours. When hypoxic exposure was prolonged to 10 days, permeability returned to the levels seen in animals maintained in room air.

Transcripts of genes that regulate vascular permeability were surveyed by qPCR from whole lung extracts. The inducible nitric oxide synthase (iNOS) and vascular endothelial growth factor A (VEGF) are expressed at the highest levels after acute hypoxia, when permeability and metastatic incidence are also increased, whereas levels of VE-cadherin transcript drop in acute hypoxia (Supplementary Fig. 2B). Arginase II expression, a HIF-2α target^7^, increases only after prolonged hypoxia. These differences are not statistically significant, illustrating the diverse contribution of different cell types to whole-lung transcript levels, likely responding in different ways to the same hypoxic challenge, and underscoring that only a subset will effectively contribute to the observed trends.

iNOS protein was shown to be visibly elevated by acute hypoxia in frozen lung sections (Supplementary Fig. 2C,D) and also in primary EC (Supplementary Fig. 2E); iNOS activity is transcriptionally regulated by HIF-1α, but not HIF-2α, and its accumulation corroborates HIF-1α activation and suggests increased permeability as a result of subsequent NO production^15^.

**Figure 2:**
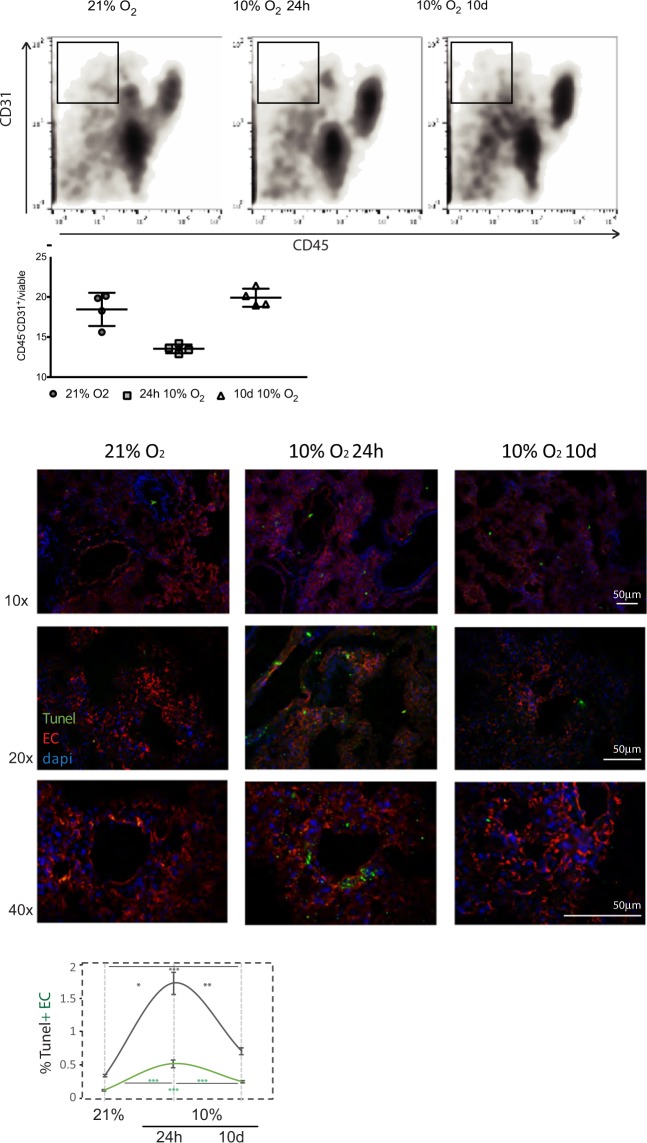
Endothelial cell population decreases with acute hypoxia exposure (**A**) Representative flow cytometry plots sorted for EC marker CD31^+^CD45^−^ viable cells are shown for each treatment and control; lung tissue was sorted immediately after hypoxia treatment (no tumour injection); graph below represents average % of viable EC (CD31^+^CD45^−^); (**B**) Representative images of TUNEL staining of frozen lung sections from mice exposed to normoxia and hypoxia, and counter-stained with vasculature marker Podocalyxin; scale bar = 50 μm; Quantification of total TUNEL staining in each group (grey line) and EC + TUNEL (green line) obtained by co-localization of TUNEL and Podocalyxin signals; values represent Avg. ± SE number of TUNEL-positive cells; images were obtained from randomly selected individual sections of a minimum of 4 mice in each treatment, at least 8 sections per animal, 3 images per section. *p < 0.05, **p < 0.005, ***p < 0.001, by Student’s *t*-test with Welch’s correction.

To evaluate potential differences in the lung microenvironment during hypoxia, different cell populations were quantified by flow cytometry of lungs harvested immediately after each treatment. A significant decrease in the relative number of EC (CD31^+^CD45^−^) is seen after acute hypoxia (Fig. 2A). An additional group of animals instead received intravenous tumour cell injections after each hypoxia pre-treatment, and were subsequently removed from the hypoxic chambers; lungs were analysed by flow cytometry only 24 h later. The decrease in CD31^+^CD45^−^ cells in this experimental group is strikingly exacerbated (Supplementary Fig. 3), suggestive of a suppression in EC proliferation, or increased cell death as part of the remodelling process^31^ which is only seen in the acute hypoxia pre-condition, but not in the chronically treated animals.

To investigate this possibility, cell death was visualised by TUNEL signal, which was increased after acute hypoxia. Figure 2B shows representative images of TUNEL-stained frozen sections at different magnifications, to illustrate changes in overall rates of cell death as well as tissue distribution of TUNEL-positive cells. Signal per section (percent TUNEL^+^ cells per field), is shown in the chart below (gray line), and EC-specific TUNEL (TUNEL + Podocalyxin, green line), shows that the pattern of cell death for EC is similar to that seen for whole lung, and correlates with the increased vascular permeability seen at this time point (Supplementary Fig. 2A).

### Selective macrophage recruitment after short and prolonged hypoxia

**Figure 3:**
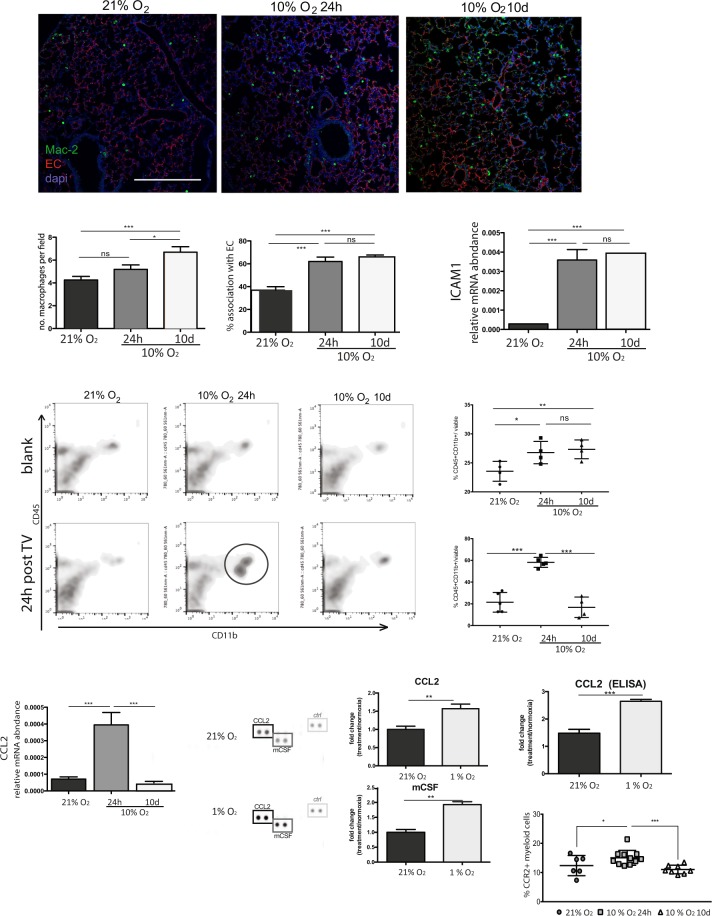
Inflammatory landscape during hypoxia exposure is distinct after short and long treatments and correlates with metastatic pre-disposition. (**A**) Representative IF images from paraffin-embedded lung sections probed for vasculature (Podocalyxin) and myeloid cells (Mac-2); scale bar = 100 μm (**B**) left, no. of Mac-2^+^ cells per field; right, % Mac-2^+^ cells associated with EC; Shown Avg ± SD, n ≥ 4 mice per treatment, 10 sections/animal; (**C**) relative mRNA levels for intercellular adhesion molecule (ICAM1) in whole lung tissue (same animals as in A and B), shown Avg ± SD; (**D**) Representative flow cytometry scatter plots of CD45^+^CD11b^+^ cells from samples obtained immediately after each treatment (blank, no tumour cell injection, top panel) or 24 h post tail vein injection of tumour cells (bottom panel). Animals are transferred to normal room air after tumour cell injection to avoid hypoxia effect on tumour cell motility; quantification is shown on the right, n ≥ 4 mice per group; (**E**) CCL2 mRNA levels in whole lung; Avg. ± SD, n ≥ 4; (**F**) Cytokine array of normoxic and hypoxic primary lung EC-conditioned medium; representative blots showing CCL2 and mCSF signal (left), quantification by densitometry, normalized to internal array controls (light grey box) on the right; Avg. ± SD, n = 3; (**G**) ELISA of CCL2 in conditioned media (same as in **F**); (**H**) %CCR2^+^ cells in myeloid population obtained by FACS of whole lung tissue after each treatment, Avg. SEM, n ≥ 6. Significance in all cases was assessed by students *t*-test with Welch’s correction, and *p < 0.05, **p < 0.005, ***p < 0.001.

Because myeloid cell infiltration is implicated in vascular remodelling and extravasation^32,33^, an antigen-presenting cell population was identified by Mac2 (Galectin-3^34^) staining, in paraffin-embedded tissue of lungs from mice exposed to different treatments (Fig. 3A). Mac2-positive cells represent a heterogeneous group of myeloid cells that can perform multiple functions. Their density appears higher in acutely hypoxic lungs and is maintained in lungs exposed to chronic hypoxia (Fig. 3B, left). Association of Mac2-positive cells (green) with the endothelium (red), known to be a key parameter in tumour cell transendothelial migration^32,35,36^, is also not different between acute and chronic hypoxia, although in both cases it is higher than in animals maintained in normoxic room air (Fig. 3B, right). Finally, whole lung mRNA levels of intercellular adhesion molecule 1 (ICAM1), involved in mediating endothelium-macrophage adhesion^37,38^, are also higher in both hypoxia treatments relative to levels seen in lungs from control animals (Fig. 3C). These data suggest an overall increase in myeloid cell infiltration in hypoxic lungs, irrespective of length of hypoxia exposure, which does not correlate with the different metastatic burden seen in each condition.

This result was validated by flow cytometry, where CD45^+^CD11b^+^ cells were gated directly from the viable cell population, after hypoxia pre-treatment (Fig. 3D, top row), and a similar result was obtained, showing an increased rate of myeloid cell infiltration in response to both acute and chronic hypoxia. Intriguingly, when lungs were harvested 24 h after tumour cell injection, there was a striking increase in CD45^+^CD11b^+^ cells in the lungs of mice pre-exposed to acute hypoxia (Fig. 3D, bottom row). This suggested the presence of a recruitment signal stimulated by acute hypoxia, but that is activated or increased in the presence of a second insult, in this case, tumour cells.

CCL2 is a known mediator of EC interaction with macrophages, and essential for metastatic success in breast cancer^35,39–41^, and we investigated whether this was the signal promoting the increase in general myeloid cell population (CD45^+^CD11^+^ cells) in that group of animals. CCL2 mRNA levels were dramatically elevated in lungs of animals exposed to acute hypoxia, but not after prolonged hypoxia (Fig. 3E); this increased expression is concomitant with the transient increase in myeloid cell retention seen in Fig. 3D (bottom panel).

CCL2 levels were also seen to increase in primary lung EC-conditioned medium (Fig. 3F), as were levels of mCSF1 (also a mediator of EC-myeloid cell communication). Levels of EC-secreted CCL2 were further verified by ELISA in the same conditioned media samples (Fig. 3G). These results suggest that CCL2, produced by EC after acute hypoxia, potentiates lung vulnerability to CTC by remodelling the pulmonary tissue microenvironment.

To confirm this, we assessed the infiltration of CCR2^+^ macrophages into the lung by flow cytometry, which is seen to increase only in animals exposed to acute hypoxia (Fig. 3H). These data show that the myeloid cells identified as Mac2^+^ (Fig. 3A) or CD45^+^CD11b^+^ (Fig. 3D), represent a heterogeneous myeloid cell population, and that EC-derived CCL2 (a HIF-1α transcriptional target^42,43^) selects for the arrest of pro-metastatic myeloid cells (CCR2^+^)^39^ in the lung during acute hypoxia. Mice were given tumour cell injections while still in the hypoxic chamber, but were returned to room air for either 24 h (to evaluate changes during early extravasation events) or 14d (to assess overt metastasis). They were returned to normoxia to circumvent the effect that continuous hypoxia would have in the movement of tumour cells themselves, or in tumour progression. This allowed observations to be attributed only to the effect of pre-existing hypoxic microenvironment. In either acute or prolonged hypoxia, the removal of the animals from the chamber post-tumour cell injection resulted in a period of reoxygenation, which will likely affect the vasculature. Nevertheless, and irrespective of the oxidative consequences derived from the experimental approach, lungs of mice exposed to prolonged hypoxia did not show selective retainment of CD45^+^CD11^+^ cells.

### EC viability and pulmonary vascular permeability are mediated by HIF

**Figure 4:**
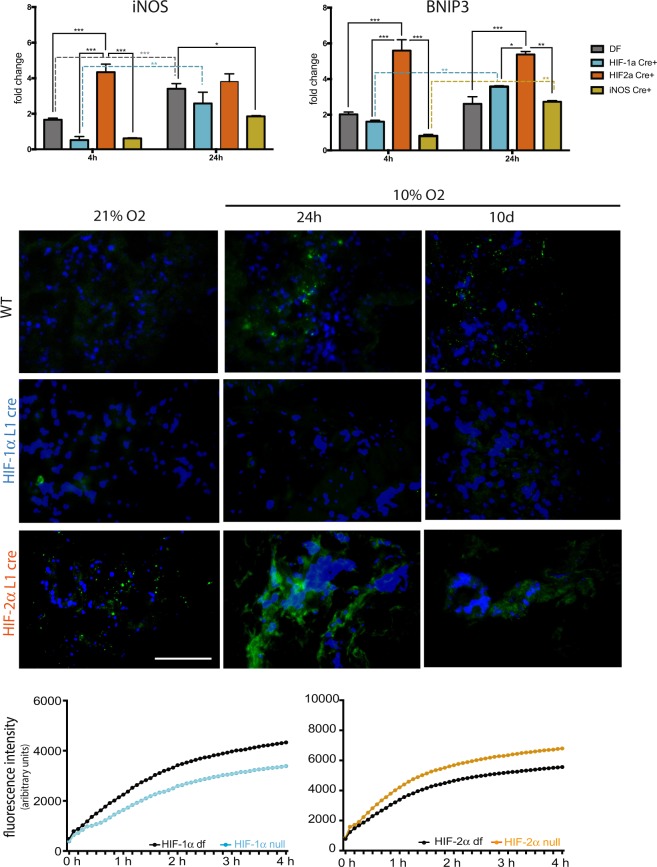
Hypoxia effects on iNOS and BNIP3 expression is HIF-α dependent. (**A**) qPCR of iNOS and BNIP3 in primary lung EC exposed to hypoxia; samples include WT (double-floxed controls, cre-negative), HIF-1α Cre^+^, HIF-2α Cre^+^ and iNOS Cre^+^ (infected *ex-vivo*), and data represents average fold change ± SEM, from combined results from two independent experiments, 3 replicates each; statistical significance was assessed ANOVA; (**B**) Qualitative assessment of iNOS signal in frozen lung sections from mice from different genotypes after different time-points; scale bar = 100 μm; (**C**) EC permeability was measured *in vitro* using freshly prepared FITC dextran (70 kDa)-containing media over primary lung EC monolayers cultured in fluoroblock inserts. Each monolayer of HIF-1α null (Cre^+^) or HIF-2α null (Cre^+^) was assessed with their respective WT controls (double-floxed, Cre^−^). Measurements are arbitrary fluorescence units that represent the amount of FITC dextran (70 kDa) that crossed the EC monolayer over time. Three biological replicates were performed per genotype.

EC viability in hypoxia is compromised relative to viability at normoxia, but Cre-mediated deletion of HIF-1α appears to protect cells from hypoxia-induced death, especially in the acute phase (4 h at 1% O_2_), as seen in Supplementary Fig. 2F. As previously shown^15^, cells lacking HIF-1α fail to accumulate iNOS mRNA after 4 h (Fig. 4A), although this is not seen after 24 h of hypoxia, suggesting an alternative and HIF-1α-independent iNOS activation. An analogous trend, albeit less marked, is seen for BNIP3 transcript in HIF-1αCre^+^ cells. iNOS and BNIP3 are known HIF-1α transcriptional targets^44^, and interestingly, both are increased in cells lacking HIF-2α after acute, but not prolonged, hypoxia.

BNIP3 drives a unique pro-apoptotic pathway, which bypasses the canonical leakage of mitochondrial cytochrome *c*^45^, and has been proposed to be activated downstream of NO^46,47^. To better understand how hypoxia/HIF-1α-induced NO accumulation might affect apoptosis, EC were harvested from animals with a conditionally targeted NOS2 (iNOS) gene^48^, which was subsequently excised *ex-vivo* by Cre-mediated recombination. We found that NOS2-null EC have impaired BNIP3 accumulation in acute, but not chronic hypoxia (Fig. 4A). It is also seen that iNOS protein levels are lower in animals lacking pulmonary EC HIF-1α^7,29^, and is highest in animals lacking lung endothelial HIF-2α (Fig. 4B).

To assess endothelial integrity, EC monolayers of different genotypes were cultured on Fluoroblock inserts, assembled in a Boyden chamber-like setting. A FITC-labelled dextran solution was loaded in the upper chamber, and the fluorescence in the lower chamber, representing the amount of dextran able to cross the endothelial barrier, was quantified in real time using an Omega plate reader (BMG Labtech). HIF-1α null endothelium (blue) is significantly less permeable than wild type (WT) (control Cre^−^ double-floxed cells, black), whereas HIF-2α null (orange) monolayers exhibit much higher permeability (Fig. 4C), suggesting HIF-mediated intercellular junction stability. The values reflect the different arbitrary fluorescent unit ranges within each plate relative to WT controls.

### Hypoxia-driven predisposition for metastasis is mediated by endothelial HIF

Animals carrying deletion of lung EC HIF-1α or HIF-2α were used to investigate *in vivo* hypoxia-induced metastasis. HIF-1αL1 and HIF-2αL1 animals, generated with specific Cre recombinase expression in the pulmonary endothelium^29^, and Cre^−^ controls were exposed to hypoxia treatments prior to tumour cell injections, as before. Metastatic burden was again assessed 14 days post intravenous injection of tumour cells.

**Figure 5:**
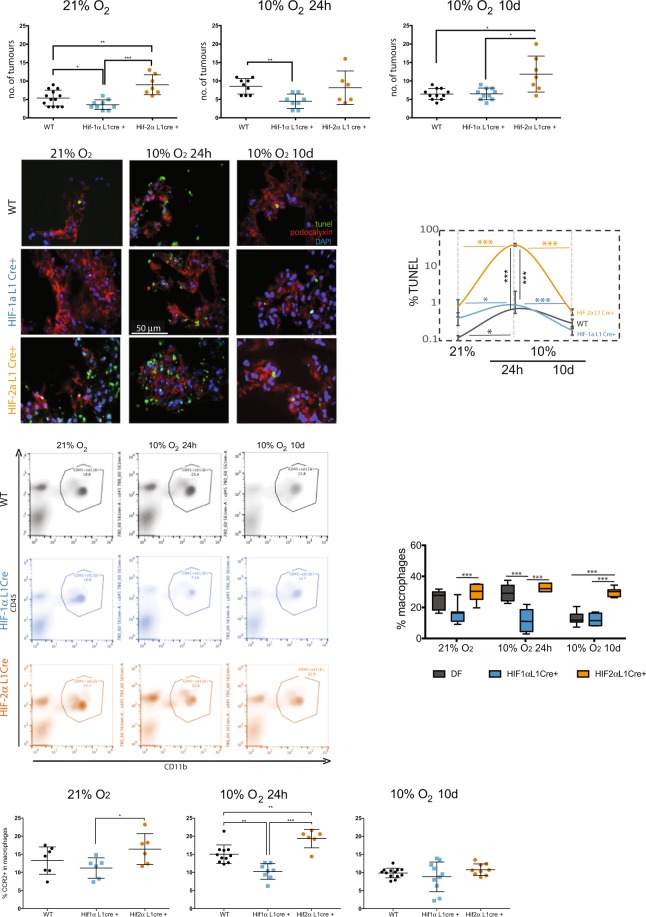
Physiological pre-disposition for metastatic colonization is dependent on lung endothelium HIF-α in isoform- and time-dependent manner. (**A**) Lung tumours per mouse were counted in H&E-stained, evenly spaced lung sections of WT animals (combined double-flox littermates, Cre^−^, black symbols), and animals lacking lung endothelium HIF-1α (L1 Cre^+^, blue symbols) or HIF-2α (L1 Cre^+^, orange symbols); n ≥ 5 animals per group, 10 paraffin- embedded sections per mouse, lungs harvested 14 days post tumour cell injection; (**B**) TUNEL staining of frozen sections of WT, HIF-1α L1Cre or HIF-2α L1Cre lung tissue after each hypoxia treatment (no tumour cell injection), bottom panel shows mean ± SEM for each genotype over time; Scale bar = 50 µm (**C**) Representative plots of interstitial macrophage population quantified by flow cytometry (after selective subtraction of non-macrophage CD45^+^ cells) in all genotypes, after each hypoxia treatment; average percent macrophages shown on the right (Avg ± SEM, n ≥ 5). (**D**) Relative proportion of CCR2^+^ macrophages in pre-conditioned lungs, obtained by flow cytometry sorting of total lung tissue from mice exposed to different hypoxia challenges and normoxic controls; WT animals (combined double-flox littermates, Cre^−^, black symbols), and animals lacking lung endothelium HIF-1α (L1Cre^+^, blue symbols) or HIF-2α (L1Cre^+^, orange symbols) were used; n ≥ 5; average SEM are shown, statistical significance was assessed by student’s *t*-tests, *p < 0.05, ***p < 0.001.

In normoxic conditions, deletion of HIF-1α (blue) results in fewer lung tumours, whereas deletion of HIF-2α (orange) leads to increased tumour burden (Fig. 5A, left). Acute hypoxia exposure does not cause increased lung tumour frequency in the HIF-1αL1 mice (blue symbols in left and middle panels), whereas control mice (black symbols) show more tumours when subjected to this same treatment (Fig. 1D). The difference in metastatic burden between WT and HIF-1α pulmonary EC deletion mice pre-exposed to acute hypoxia is thus significantly exacerbated. Interestingly, acute hypoxia does not further increase metastasis in HIF-2α pulmonary EC deletion animals compared to normoxic controls (orange symbols, left and middle panels), and as such there is no significant difference in tumour burden between those and WT controls. However, HIF-2αL1 animals pre-exposed to chronic hypoxia show more lung tumours when compared to the other genotypes in the same pre-treatment, and at this time-point there is no difference between the metastatic burden found in HIF-1αL1 and WT.

When assessing tumour areas within tumour-containing sections in the hypoxia treated groups (either acute or chronic), the differences between genotypes are subtle (Supplementary Fig. 4). However, the proportion of sections containing tumours, consistent with overall tumour burden, is lower in animals lacking endothelial HIF-1α in all pre-treatments, especially after acute hypoxia (Supplementary Fig. 4B). This argues for a significant role of this isoform in disrupting endothelial barrier function during the initial phase of the vascular hypoxia response, and in promoting the frequency of extravasation events, but not necessarily the proliferation of extravasated cells or the expansion of secondary tumours.

All genotypes have more TUNEL^+^ cells after acute hypoxia, and in all cases these decrease after chronic hypoxia exposure. Additionally, HIF-2αL1 mice have consistently higher percentages of TUNEL^+^ cells (Fig. 5B) than the other genotypes, consistent with viability pattern seen in cultured EC (Supplementary Fig. 2F). However, unlike what was seen in isolated EC, HIF-1αL1 lungs appear to have slightly higher rates of EC cell death than WT (Fig. 5B).

Co-localization of TUNEL and Podocalyxin signals is shown in Table 1. This table summarises the proportion of EC found within the total TUNEL^+^ cells, not the absolute number of TUNEL^+^EC. In the group treated for acute hypoxia (24 h), HIF-1αL1 lungs have an average 15.5% TUNEL^+^ EC; this is considerably lower than the rates of EC cell death seen in wild-type animals (27.9%). HIF2α-L1 animals show a much higher rate of EC cell death, with an average 49.4% of TUNEL^+^ EC co-staining. This discrepancy is not seen in either of the other treatments, corroborating a HIF-1α-driven cell death that occurs only during an acute response to hypoxia. HIF-2αL1 mice have constitutively higher TUNEL^+ ^EC than other genotypes under all conditions, and thus the slightly lower proportion of dead EC in the 10d hypoxia group in fact represents overall more TUNEL^+^ EC, because there is higher TUNEL signal overall (approximately 2-fold higher than seen HIF-1αL1 animals, Fig. 5B).

**Table 1:** Average co-localization of TUNEL and Podocalyxin signal in frozen lung tissue following hypoxia pre-conditioning (n ≥10).

Treatment	WT		HIF-1αL1		HIF-2αL1	
	Avg co-localization	*SEM*	Avg co-localization	*SEM*	Avg co-localization	*SEM*
21% O_2_	0.311	*0*.*0310*	0.367	*0*.*0719*	0.378	*0*.*0929*
10% O_2_ 24 h	0.279	*0*.*0421*	0.155	*0*.*0311*	0.494	*0*.*0437*
10% O_2_ 10d	0.388	*0*.*0373*	0.446	*0*.*0541*	0.409	*0*.*0716*

### Endothelial response to hypoxia affects the lung inflammatory milieu

Numerous studies have reported the role of macrophages in tumour cell extravasation^32,49–51^, as well as in vascular remodelling^49^. Thus, it was essential to understand if the rates of inflammation in response to acute and chronic hypoxia in mutant lungs were different from those seen in WT controls; this was analysed by observing the CD45^+^CD11b^+^ cell population. Unlike in Fig. 3D, the gate was applied after selective subtraction of other cells from the myeloid lineage (CD45^+^) to more accurately quantify macrophage infiltration^52^.

In WT animals, a higher number of macrophages was found in lungs after 24 h of hypoxia (Fig. 5C, grey panels), correlating with increased metastatic incidence (Fig. 1D). HIF-1αL1 mice (blue) showed a constitutively lower proportion of these cells, whereas HIF-2αL1 (orange) have consistently higher numbers of lung macrophages: HIF-2αL1 animals exhibit constitutive inflammation. The proportion of CCR2^+^ cells within this macrophage population is lower in HIF-1αL1 mice exposed to acute hypoxia (24 h) (Fig. 5D), in correlation with the lower level of metastatic events observed in this genotype (Fig. 5A). Following chronic hypoxia, there are no differences in the levels of CCR2^+^ macrophages between genotypes, consistent with CCL2 induction forming part of an acute and HIF-1α-mediated response.

## Discussion

Exposure to hypoxia involves adaptive responses that evolve over time. The remodelling processes that occur in lungs when exposed to transient or persistent changes in oxygen availability cause changes in immediate and long-term organ physiology and adaptive strategies to subsequent stimuli^7^. These rely initially on a shift to glycolytic metabolism^10,53^, which is mediated by HIF-1α. HIF-1α also regulates cell fate via transcriptional activation of apoptotic mediators including BNIP3 and p53^54^. The transient increase of HIF-1α seen in whole lung tissue following acute hypoxia correlates with increased tumour numbers, transient increases in pulmonary microvessel permeability and iNOS expression (Fig. 5 and Supplementary Fig. 2B–E). Deletion of HIF-1α results in decreased iNOS and BNIP3 transcript levels in cultured EC, less paracellular permeability (Fig. 4), and fewer apoptotic EC in lungs after exposure to acute hypoxia (Fig. 5). Even though HIF activation occurs in multiple cell types within the lung, the experiments performed using conditional deletion of HIF in pulmonary endothelium indicate a substantial role of these transcription factors, specifically in the microvasculature, in the metastatic process.

Lungs lacking endothelial HIF-2α, however, have more TUNEL^+^ EC, and higher iNOS and BNIP3 mRNA expression (Fig. 4A), in line with previous reports of NO-potentiation of BNIP3 signalling^46^. HIF-2α is an essential regulator of endothelial barrier function, as shown in brain microvascular EC exposed to chronic hypoxia^55^. Deletion of endothelial HIF-2α has also been shown to prevent recovery from kidney injury and inflammation^4,56^, and has been proposed to be a transcriptional co-activator of VE-cadherin in a hypoxia-independent fashion^5^. EC are especially vulnerable to anoikis when compared to other cell types^57^, and the increased cell death seen in cultured pulmonary HIF-2α null EC is likely a result of deficient EC-EC cell adhesion; this hypothesis is supported by the higher paracellular permeability seen in these monolayers (Fig. 4C).

iNOS accumulation and subsequent NO production are critical factors in mediating endothelial permeability and cell death, which can, to a large extent, explain the increased metastatic burden in conditions where HIF-1α activity is highest: HIF-1αL1 animals have lower levels of iNOS, as expected^44^, whereas those lacking lung endothelium HIF-2α (HIF-2L1) have much higher iNOS transcript levels. This is seen mostly at earlier hypoxia time-points.

**Figure 6:**
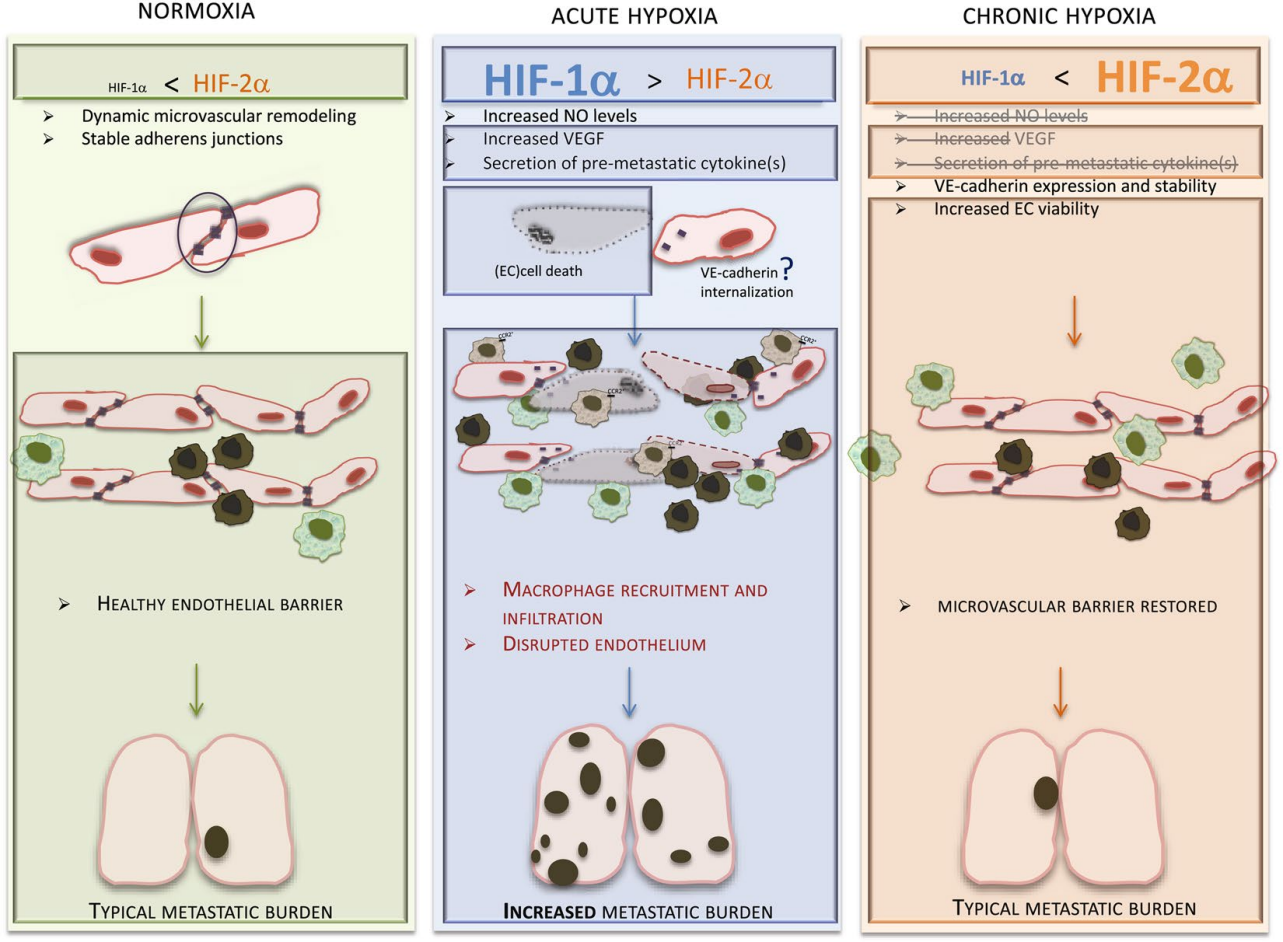
Effect of hypoxia in HIF-α isoform activation and endothelial barrier remodeling. In this model, we integrate the findings of this study (in shaded enclosed boxes) with previously reported and/or hypothesised processes in EC stability and metastasis. Endothelial HIF-1α levels are negligible in normoxic lung environment (left), whereas HIF-2α is constitutively present; this is essential for vessel stability^4^, microvascular perfusion^56^ and endothelial barrier function (as shown in^15^ and Fig. 4). During the initial stages of hypoxia response (middle panel), and as part of the vascular remodelling process^23,45^, HIF-1α levels increase dramatically in EC, largely surpassing those of HIF-2α, and predictably results in increased NO production^7,15^, VEGF levels, apoptotic and endocrine signals (Figs 3 and 4), promoting infiltration and retention of inflammatory cells (Figs 3 and 5), specifically pro-metastatic CCR2^+^ macrophages^39,41^ (Fig. 5). The combined effects of acute hypoxia result in compromised endothelial integrity and inflammation, which should facilitate extravasation, and promote metastasis. With prolonged exposure to hypoxia (right panel), levels of HIF-2α increase to higher than those of HIF-1α, reinstating the balance observed in normoxia. Increased levels of HIF-2α should restore adherens junctions^4^ and monolayer stability, and the absence of HIF-1α results in less cell death and removal of CCL2 recruitment signal. Deletion of endothelial HIF-2α results in compromised integrity and likely the microvasculature’s ability to regenerate, leading to increased metastatic incidence (Fig. 6).

In Fig. 6, we show a model to incorporate the findings shown in this study (within shaded boxes) with previously reported data: we see differential proportion of HIF isoforms in lung endothelium depending on the oxygen levels and duration of hypoxia. The increased permeability of lung endothelium, seen after acute hypoxia, results from an increase in HIF-1α-mediated cell death; WT lungs recover after prolonged hypoxia exposure, likely via incremental activation of HIF-2α, but in the absence of this isoform endothelial integrity is compromised and appears unable to recover. This suggests that EC viability^58,59^ is compromised in HIF-2aL1 lungs as a result of deficient monolayer stability (possibly disruption of adherens junctions^4,5^) and increased anoikis^60^. Consequently, the interrupted microvessel barrier will facilitate extravasation events and lead to the higher metastatic burden seen in the absence of endothelial HIF-2α.

Also illustrated in Fig. 6 is that increased HIF-1α levels in hypoxic lungs correlate with macrophage infiltration; this has been shown to promote tumour growth and metastasis^22,27^; our results suggest that prolonged exposure to hypoxia allows vascular regeneration, in agreement with previous reports that this is a process dependent on HIF-2α activation^4,7,28,61^. We also show that the endocrine function of lung EC changes throughout the duration of the hypoxic stimulus, in turn affecting the recruitment and arrest of myeloid cells to the lung (Figs 4 and 5). Upon exposure to acute hypoxia, there is a striking increase in CCL2, likely downstream of HIF-1α activation^42,43^, and an effective recruitment and arrest of CCR2^+^ macrophages from an already exacerbated myeloid cell population (Fig. 3). CCR2^+^ macrophage infiltration is not seen in HIF-1αL1 animals, and is conversely seen at much higher levels in HIF-2αL1 animals only after acute hypoxia; this correlates with highest CCL2 (and HIF-1α) signals (Figs 1 and 5). We anticipate that the constitutively high density of macrophages seen in the EC null HIF-2α mice is a result of high rates of EC death in the lungs of these animals.

Multiple factors affect extravasation and tumour cell survival post extravasation, including macrophage assistance during transendothelial migration, matrix remodelling and post-extravasation vascularization of incipient tumour. This study demonstrates that EC mediate a tissue’s predisposition for metastatic colonization, including the selective recruitment of myeloid cells. This effect is seen specifically in changes in endothelial viability, integrity and endocrine function, downstream of activation of specific branches in the HIF pathway. Multiple cells may respond to the HIF-stabilizing stimulus, also in a time-dependent manner (Fig. 1C). Our observations validate the contribution of the endothelium-derived HIF, both in constitutive and adaptive responses, to the metastatic potential of the lung. Importantly, similarly EC-dependent remodelling phenomena are likely to occur in other pre-metastatic organs^62^.

Our experimental approach used hypoxia as a driver for differential HIF-isoform stabilization, and as a model to understand the role of HIF in the pre-metastatic microenvironment. This study does not include the effects of hypoxia in other cell types within the lung, nor the systemic effects of atmospheric hypoxia in cancer progression and dissemination. Additionally, this model does not account for the fact that HIF isoform stabilization is initiated by signals other than hypoxia^17,23^, especially in the context of cancer^63^ and therapy^12,64^, and those limitations should be addressed in the future. However, with the use of lung EC-specific deletion of HIF-1α and HIF-2α, we have demonstrated and substantiated the non-redundancy of these transcription factors in endothelial barrier function, and the relevance of the endothelium in mediating pre-metastatic organ remodelling. The understanding of the molecular events downstream of cell type- and organ-specific HIF-α activation has powerful implications in the ability to pre-empt distant organ vulnerability to metastatic disease, and potentiate the development of improved diagnostic and therapeutic approaches for aggressive cancer types.

## Methods

### Please refer to Supplementary file for extended methods

#### Animal experiments

Mice were bred and housed in specific pathogen-free conditions according to ethical regulations of the UK Home Office and the University of Cambridge. All animals are Bl57/6, whether wild type or homozygous for lox p-flanked HIF-1α or HIF-2α. Specific deletion of HIF-1α or HIF-2α in pulmonary EC was generated by introducing the specific promoter L1^65^ into the homozygous floxed animals (DF). Crosses were performed using DF females and DF males carrying one copy of L1Cre allele (heterozygous), such that only 50% of progeny would express the Cre recombinase, and the remaining animals would be Cre-negative, and used as WT littermate controls. Cre-mediated deletion of HIF-1α allele^65^, shown in Supplementary Fig. 5A, has been shown to be consistently above 90%, and that of the HIF-2α allele^66^ was recently shown to be 80%^7^. Deletion was quantified by isolation of genomic DNA from endothelial cells from double-floxed male mice, exposed to adenoviral Cre infection, and normalized to DNA sequence of wild-type genes (with no lop-P sites, in this case, VEGF), and compared to cells trated with Adenovirus expressing b-galactosidase as controls for viral infection. Supplementary Fig. 5B,C show demonstrative IF images of L1 Cre animals, and qualitative effect of EC HIF-deletion in the detection of each isoform. Male mice were exposed to 10% O_2_ for either 24 h or 10d, in chambers with controlled humidity and CO_2_; control animals were maintained in normal atmosphere.

#### Metastasis assay

5 × 10^5^ LLC^GFP^ cells were injected intravenously in control or hypoxia pre-treated animals. Animals were removed from hypoxia chambers after tumour cell injection, to avoid interference of hypoxia with tumour cell behaviour, and evaluate exclusively the effect of pre-existing conditions. Lungs were collected either 24 h after tumour cell injection, to assess immediate changes in lung milieu, or 14d post-injection, for long term changes and quantification of tumour seeding. Fresh tissue was harvested for flow cytometry, snap-frozen for further processing, or paraffin-embedded for histological assessment of tumour burden.

#### Pulmonary vascular permeability

Evans Blue (EB) dye (50 mg/Kg) was injected intravenously into control and hypoxia pre-conditioned animals; Bronchoalveolar lavage was collected in 1 mL of PBS 20 min later, and the concentration of EB leaked from the vasculature into the bronchoalveolar space was quantified against a standard curve at 540 nm.

#### Flow cytometry

Lung tissue was digested and filtered into single cell suspensions, followed by incubation with fluoro-conjugated antibodies. Data was acquired on Fortessa (BD Biosciences) and analysed on FlowJo (FlowJo, LLC). Detailed protocols and gating strategy can be found in the Supplementary Extended Methods section.

#### Immunofluorescence

Lung tissue was collected immediately after hypoxia treatments into OCT compound and promptly frozen, without perfusion or inflation, to avoid affecting the stability of the HIF-a protein signal and the integrity of the endothelium itself. Primary antibody incubations (HIF-1α -NB100-105^67^, 1:50; HIF-2α - NB100-132^68^, 1:150; iNOS, SC-651; Podocalyxin (EC counterstain^69^), R&D Systems AF1556, 1:200) occurred O/N at 4 °C, and fluoro-conjugated secondary antibodies (Alexa Fluor 488, Thermofisher Scientific, or Alexa Fluor 647, Abcam) for 1 hour at RT. Thermofisher- Click-iT® TUNEL Alexa Fluor® 488 Imaging Assay (#C10245) was used with slight modifications to original protocol, including additional blocking steps. In frozen tissue sections, Podocalyxin proved the most stable stain for EC; in Supplementary Fig. 5, we show vascular IF with multiple markers (Supplementary Fig. 6A), and that Podocalyxin stain overlaps with that of CD31 in these samples (Supplementary Fig. 6B), confirming the specificity. This was true especially when vascular stain was performed in combination with other primary antibodies or dyes (such as HIF-α or TUNEL). Animal tissue was analysed in 3 images per section, ≥ 8 sections/animal, for a minimum of 5 animals per group, per treatment. Primary lung EC used for imaging were cultured in gelatine-coated glass slides and fixed immediately after hypoxia treatments; 4 slide chambers per treatment, per group were used, and a minimum of 4 images/chamber were obtained for analyses. All images were obtained with a Leica fluorescence microscope or Leica SP5 confocal microscope using a 40x or 63x Oil Immersion objectives. Images were acquired through LAS (Leica) and analysed using ImageJ^70^.

#### qPCR

abundance of specific gene transcripts were performed by standard qPCR and normalised to β*-ACTIN*. Primers for iNOS, VEGF, Arginase II and β-actin were reported elsewhere^15^, BNIP3Fwd:GACGAAGTAGCTCCAAGAGTTCTCA, and BNIP3Rev: CTATTTCAGCTCTGTTGGTATCTTGTG; VE-Cadherin primers were obtained from Qiagen (Cat. No. QT000110467). Data are presented as average fold-change ± SD, unless otherwise stated. Significance was assessed by Student’s *t-*tests with Welch’s correction or ANOVA, as stated in each figure.

#### Cell culture

EC were isolated from homozygous floxed animals, and cultured as previously described^15^. Cre-Recombinase Gesicles (Clontech, cat. No. 631449) were used for *ex-vivo* gene deletion.

#### *In vitro* permeability assay

Endothelial integrity was measured using real-time imaging of FITC Dextran 70 KDa that permeated through an endothelial monolayer plated onto Fluroblok inserts (light-blocking PET, Corning), placed in 24-well companion plates (Falcon, Cat # 353504), in Boyden chamber-like set up. Readings were taken every 5 minutes for 4 hours.

#### Western blotting

Whole protein extracts were prepared in RIPA buffer containing protease inhibitors as per suppliers instructions (Roche, 11697498001), and nuclear protein extracts were obtained using NE-PER nuclear and cytoplasmic extraction reagents (ThermoScientific, cat. No. 78833). Samples were resolved in 3–8% Tris Acetate SDS gels (Life Sciences, EA0375BOX) and transferred to PVDF. Primary antibodies were used at 1:1,000 (HIF-1α, NB-100-049, iNOS, SC-651) or 1:500 dilutions (HIF-2α, R&D AF2997), and detected following incubation with HRP-conjugated secondary antibodies and ECLPlus chemiluminescence (Amersham). Image capture and quantification were performed using FusionFX (Vilber) using default exposure for optimal signal detection, and statistical analysis in all cases was done using unpaired Student’s *t-*test with Welch’s correction after normalizing target band intensity to that of β-actin control in the same sample. Original full scans of gels cropped and used in main figures can be found in Supplementary Figs 7, 8 and 9.

#### Statistical analyses

were performed using Prism 7 software, and statistical tests used are referred to in figure legends and supplementary materials.

## Ethical Approval

The study was conducted within the ethical principles of the Animal Welfare Act 1986, approved by the UK Home Office, and the Animal Welfare Ethical Review Body (AWERB) of the University of Cambridge, and within the authorities granted by the Project Licence ref. 80/2565, held by RSJ.

## Supplementary information

Supplementary Material

## Data Availability

Most data generated or analysed during this study are included in this published article and supplemental files. Additional information or specific requests are available from the corresponding author on reasonable request.
