# Author Correction: Acute and chronic hypoxia differentially predispose lungs for metastases

**DOI:** 10.1038/s41598-020-58616-0

**Published:** 2020-01-28

**Authors:** Moritz Reiterer, Renato Colaço, Pardis Emrouznejad, Anders Jensen, Helene Rundqvist, Randall S. Johnson, Cristina Branco

**Affiliations:** 1 Queen’s University Belfast, Centre for Cancer Research and Cell Biology, Belfast, UK; 2 Department of Physiology, Development and Neuroscience, University of Cambridge, Cambridge, UK; 3 Karolinska Institutet, Stockholm, Sweden

Correction to: *Scientific Reports* 10.1038/s41598-019-46763-y, published online 15 July 2019

In Figures 1–5, the panels are not labelled. The correct figures appear below.

**Figure 1:**
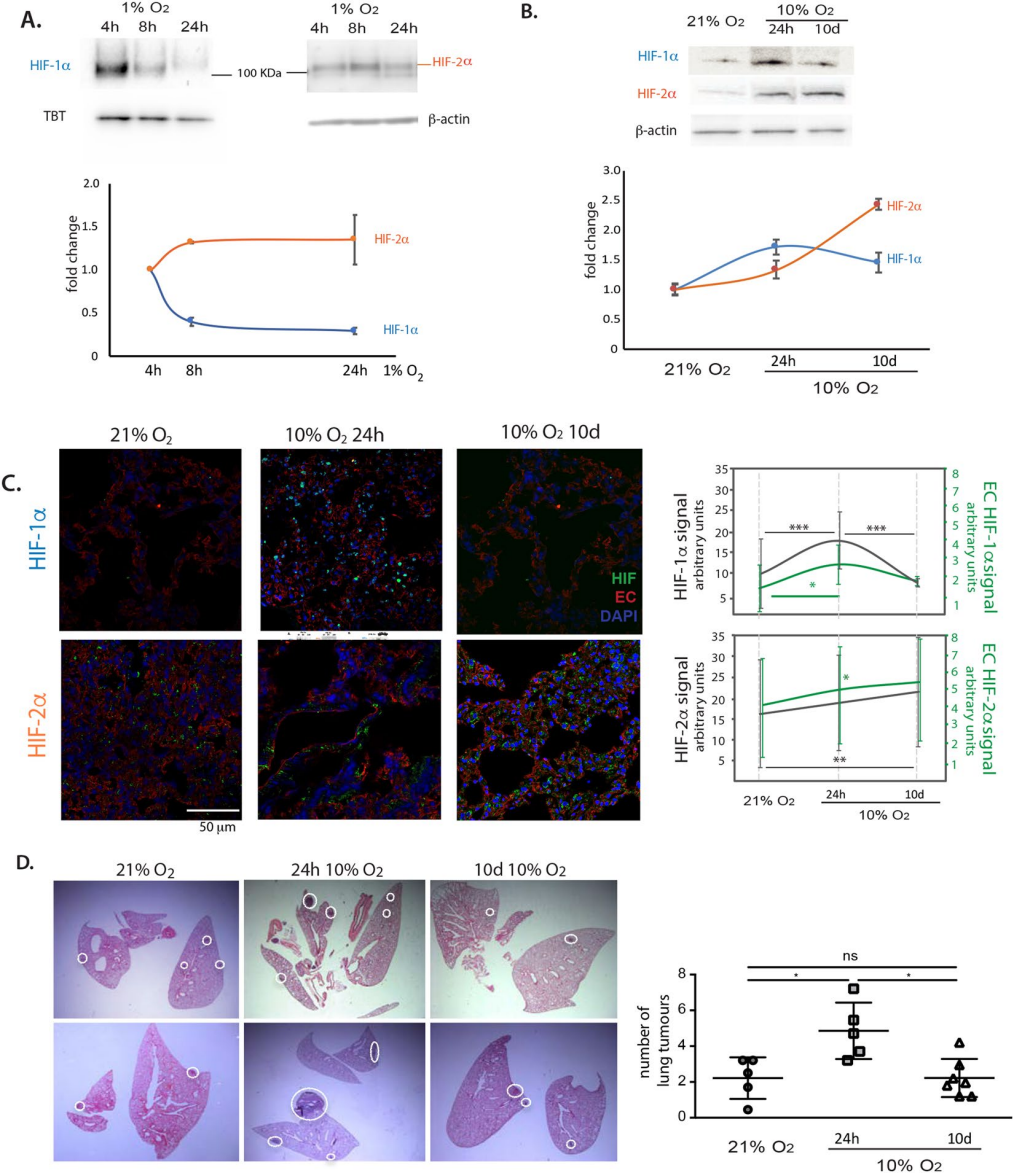
.

**Figure 2:**
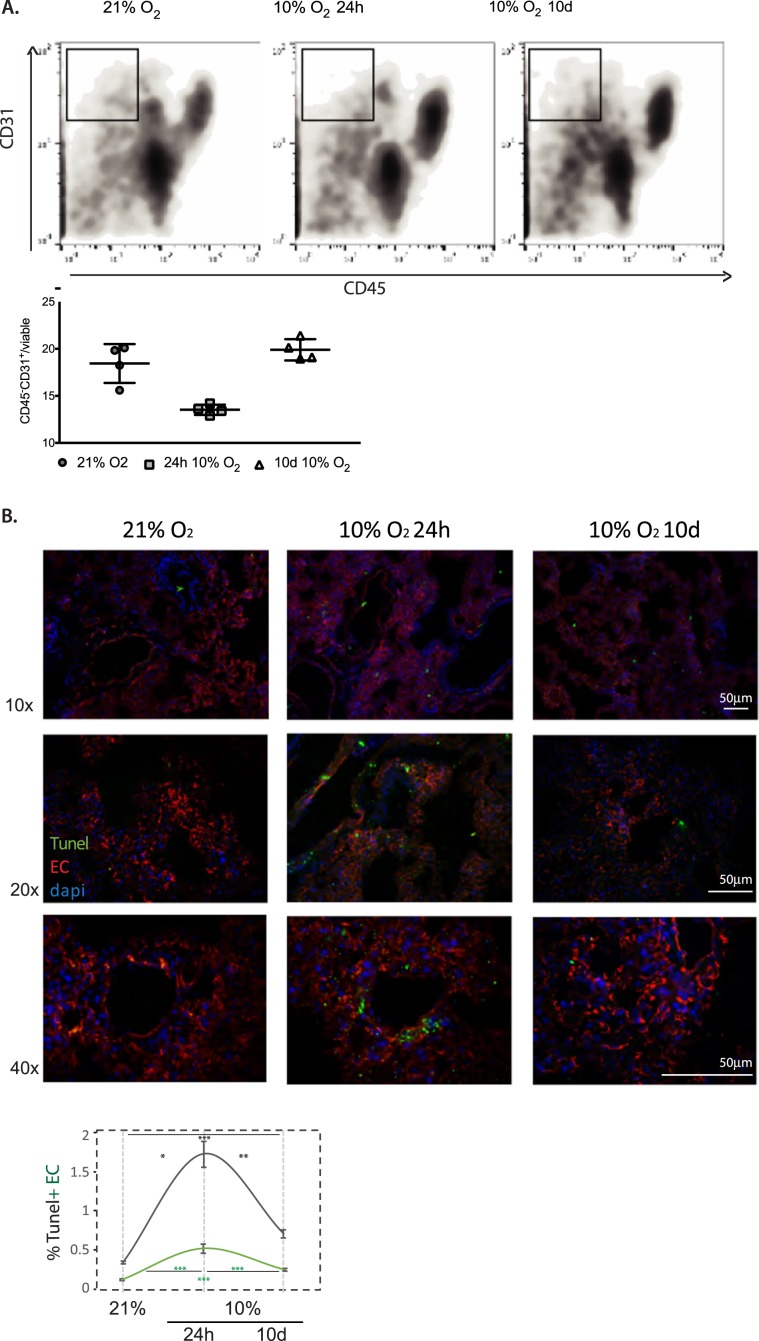
.

**Figure 3:**
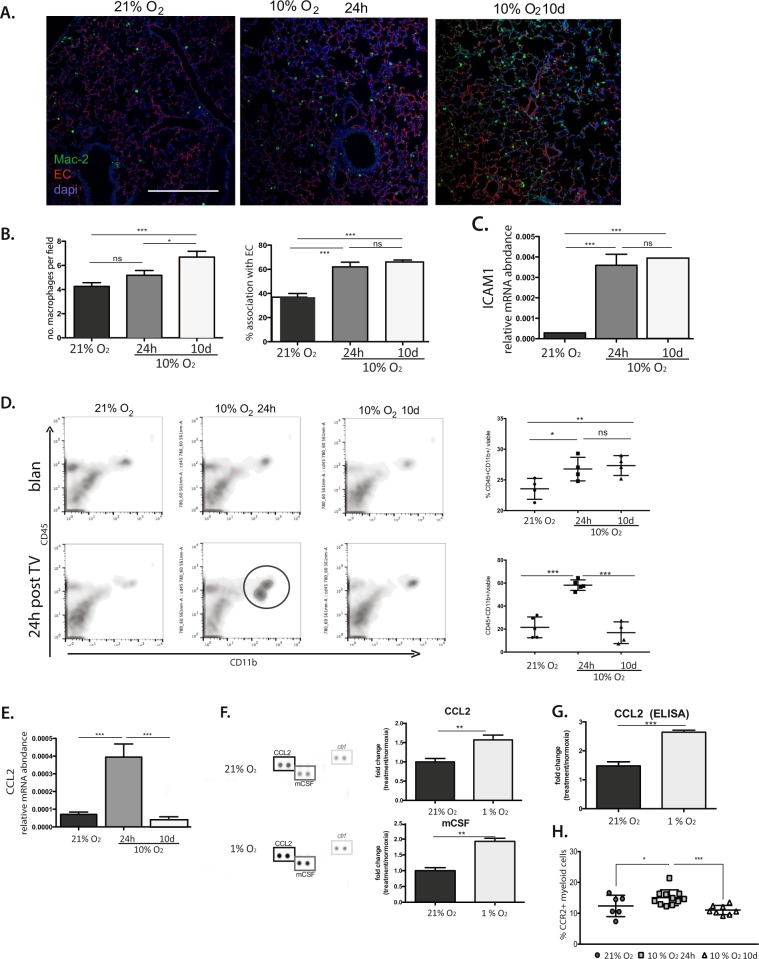
.

**Figure 4:**
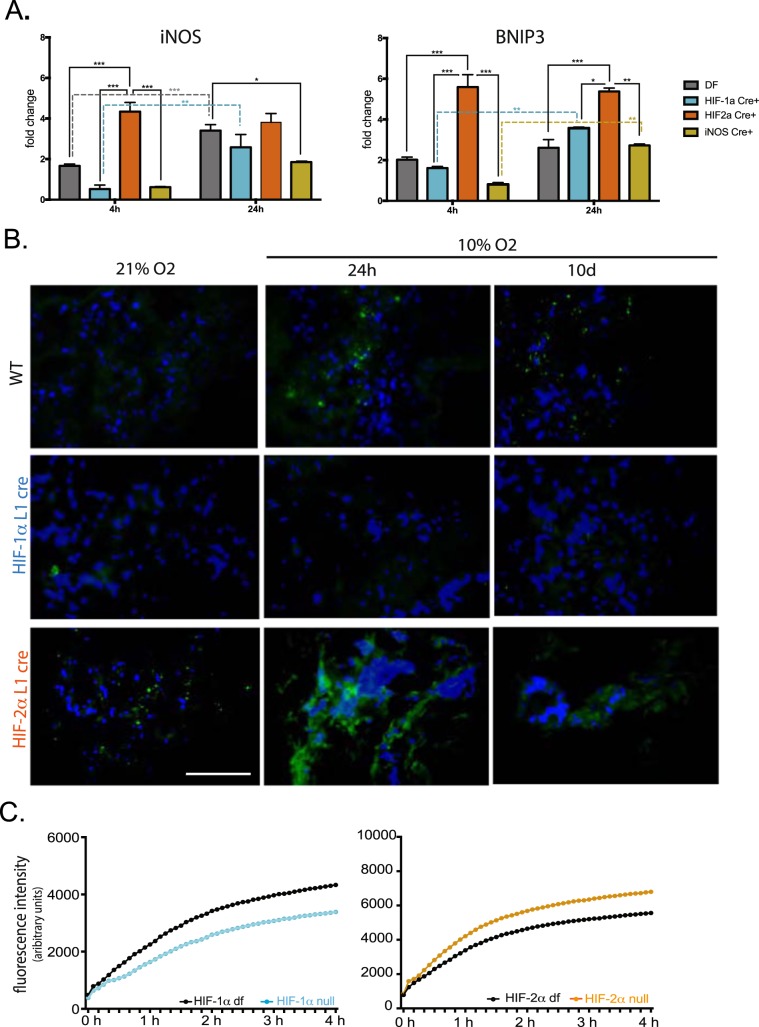
.

**Figure 5:**
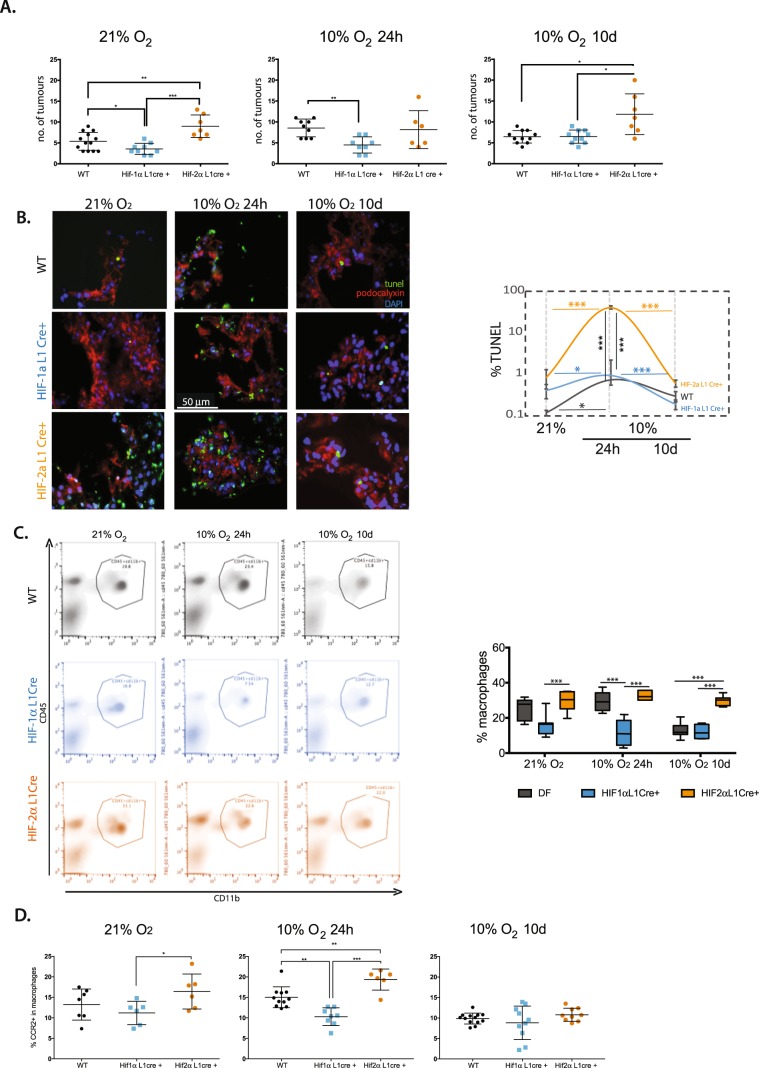
.

