# Supplementary material for: Acute and chronic hypoxia differentially predispose lungs for metastases
Source: Sci Rep. 2019 Jul 15;9:10246. doi: 10.1038/s41598-019-46763-y (PMC6629695; doi:10.1038/s41598-019-46763-y)
Supplement: Supplementary file 1 — Supplementary Material [file 41598_2019_46763_MOESM1_ESM.pdf]

Acute and chronic hypoxia differentially predispose lungs for metastases

Moritz Reiterer, Renato Colaço, Pardis Emrouznejad, Anders Jensen, Helene Rundqvist, Randall S. Johnson, Cristina Branco

Supplementary Material

Supplementary Fig. 1 - Hypothesis and Experimental Approach

Supplementary Fig. 2 – Hypoxia-induced endothelial cell death is HIF dependent

Supplementary Fig. 3 – (accessory to Figure 2) CD31+ cells decrease after acute hypoxia

Supplementary Fig. 4 - (accessory to Figure 4) E ndothelial HIF-mediates metastatic burden

Supplementary Fig. 5 – HIF-1α deletion efficiency and representative IF for WT and L1 lungs

Supplementary Fig. 6 - Endothelial cell staining validation

Supplementary Fig. 7 – Whole membrane scans of Western blot images for HIF-1α and respective loading controls, presented in Fig.1A, main text, and other replicates.

Supplementary Fig. 8 – whole membrane scans of Western blot images for HIF-2α and respective loading controls, presented in Fig.1A, main text, and other replicates.

Supplementary Fig. 9 - whole membrane scans of Western blot images for HIF-1α and HIF-2α and respective loading controls, presented in Fig.1B, main text**.**

Extended Methods

Supplementary Fig. 1

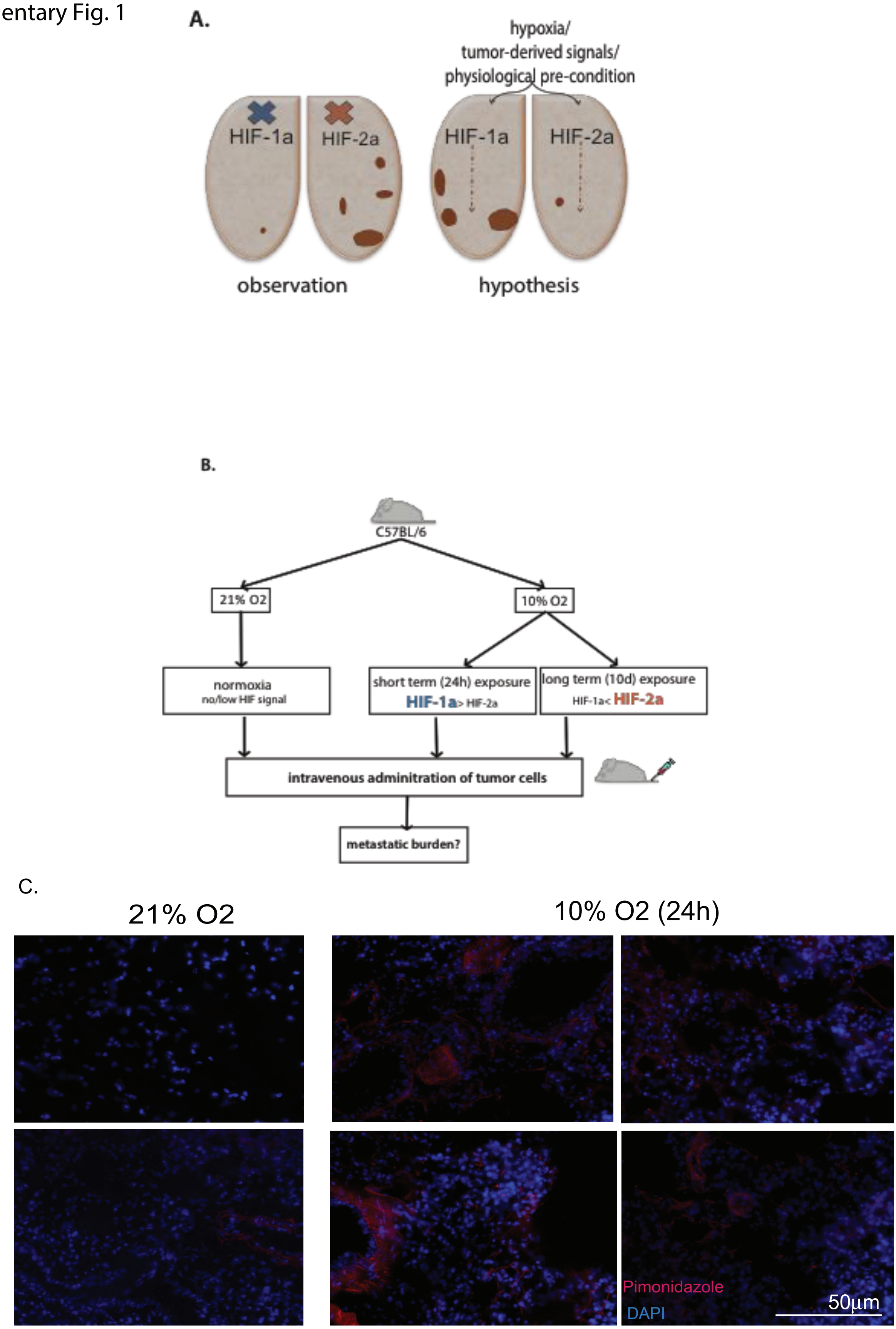

Hypothesis and Experimental approach. (A) Upon the initial observation that deletion of HIF-1a resulted in less metastasis, and deletion of HIF-2 resulted in increased metastatic burden (Branco-Price et al., 2012, left), we hypothesize that the stabilization of HIF-1 or HIF-2 would result in the opposite phenotype (right panel); (B) Using WT animals, we pre-conditioned mouse lungs using short and long hypoxia exposure to selectively stabilize HIF-1α or HIF-2α, respectively, prior to intravenous injection of tumour cells (5 x 105 LLC via TV), assess tumour burden; time points for optimal preferential proportion of specific isoforms determined in prior pilot experiments; (C) confirmation of tissue hypoxia was done by Pimonidazole staining (red) of lung tissue from animals exposed to 24h of 10% O_2, or controls kept in normal atmosphere. Multiple representative images are shown.

Supplementary Fig. 2

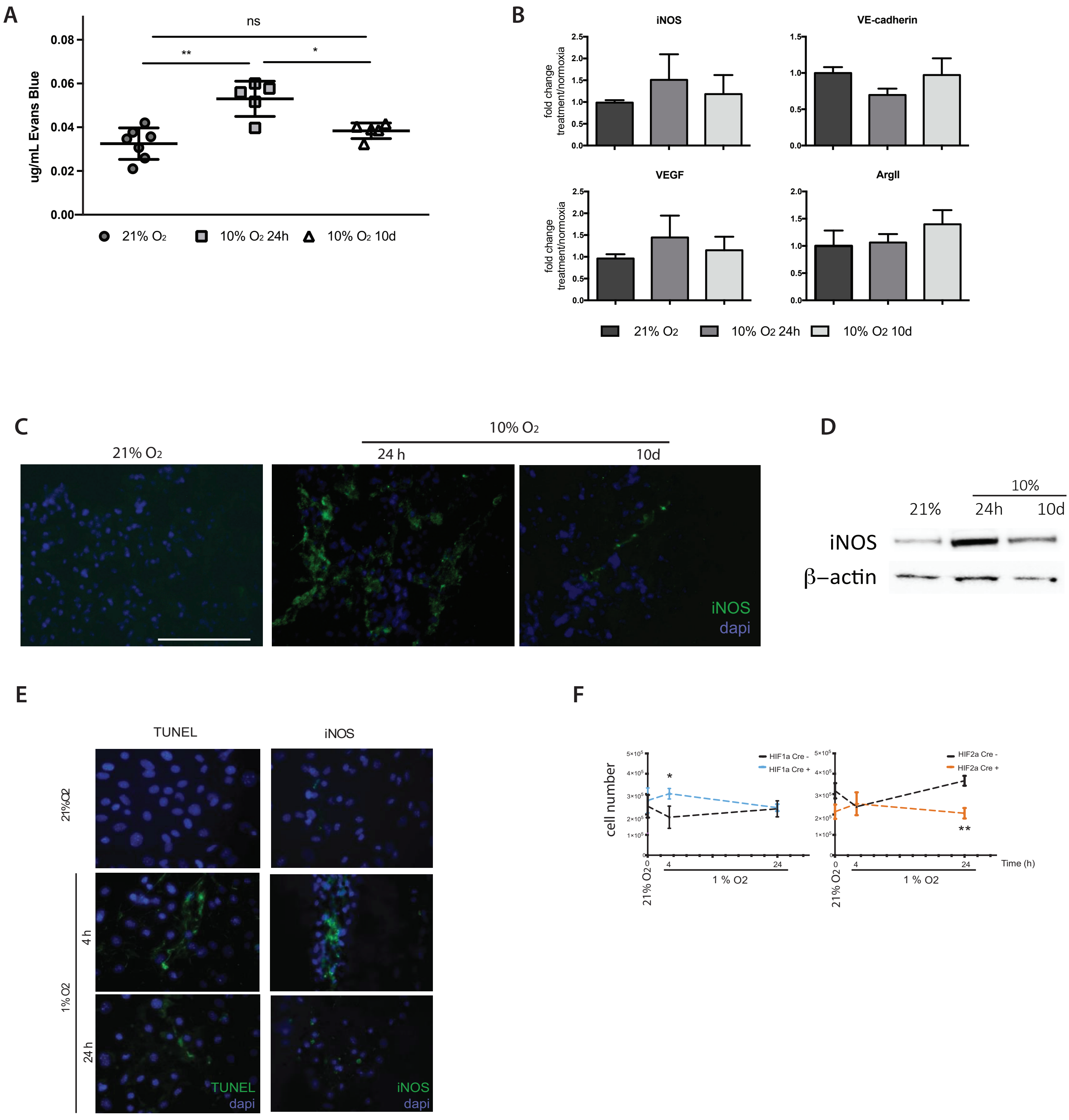

Hypoxia transiently induces vascular permeability, correlating with transient accumulation of iNOS mRNA and protein levels. (A) Lung vascular permeability was assessed by quantification of Evans Blue in Bronchoalveolar lavage. Data is average +/- SD, n > 5 animals per group. Significance was assesed by Student's t-test with Welch's correction, and * p<0.05, ** p<0.01; (B) qPCR of whole lung tissue is shown for transcripts encoding proteins involved in modulation of vascular permeability: iNOS, VEGF and VE-cadherin, and ArgII, as a surrogate marker for HIF-2   activation. Data is average fold change of signal of treatment to control (normalised to   -actin), +/- SE; (C) iNOS signal was detected by IF in frozen lung sections or animals exposed to acute or prolonged hypoxia as a qualitative representation of protein levels,   scale bar 100 μm; (D) Western Blot of whole lung protein extracts of animals exposed to normoxic or hypoxic atmosphere, probed for iNOS protein; (E) TUNEL staining and immunofluorescent detection of iNOS levels in primary EC fixed after acute (4h) and prolonged (24h) hypoxia (1% O2); (F) viability if HIF-1α null(left) or HIF-2α null (right) EC and their respective WT(double-floxed, cre-negative) controls in normoxia and after 4h and 24h of hypoxia; statistical significance was assessed by Student's t-test between genotypes for each time point, *p<0.05, ** p<0.01.

Supplementary Fig. 3

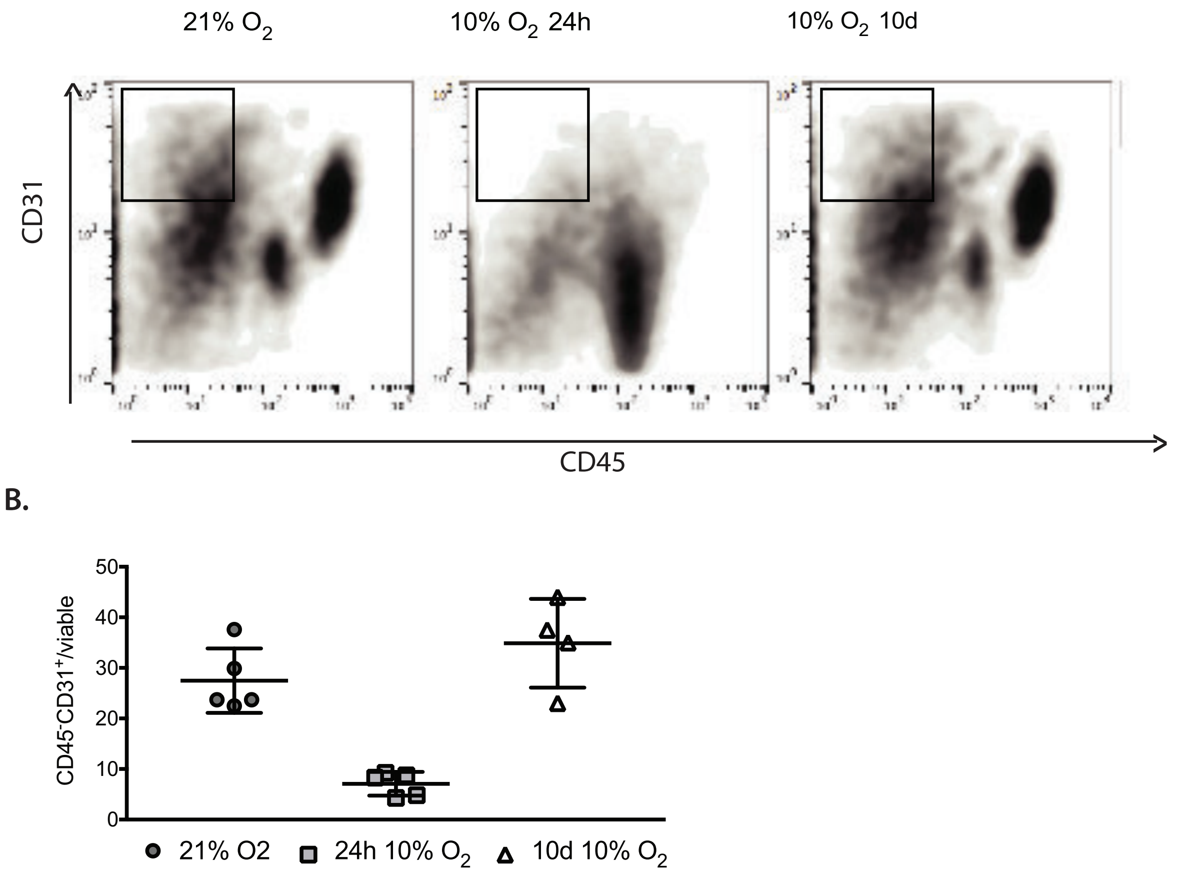

Viable endothelial cell numbers transiently decrease upon acute hypoxia exposure. Representative flow cytometry plots for identification and quantification of EC ($CD31^+CD45^-$) in whole lung tissue exposed to normoxia or hypoxia followed by tumour cell injection (via tail vein). Upon tumour cell injection, animals were removed from hypoxia chambers and lung tissue harvested 24h later. Viable cells are shown for each treatment and control; graph below represents average % of viable EC.

Supplementary Fig. 4

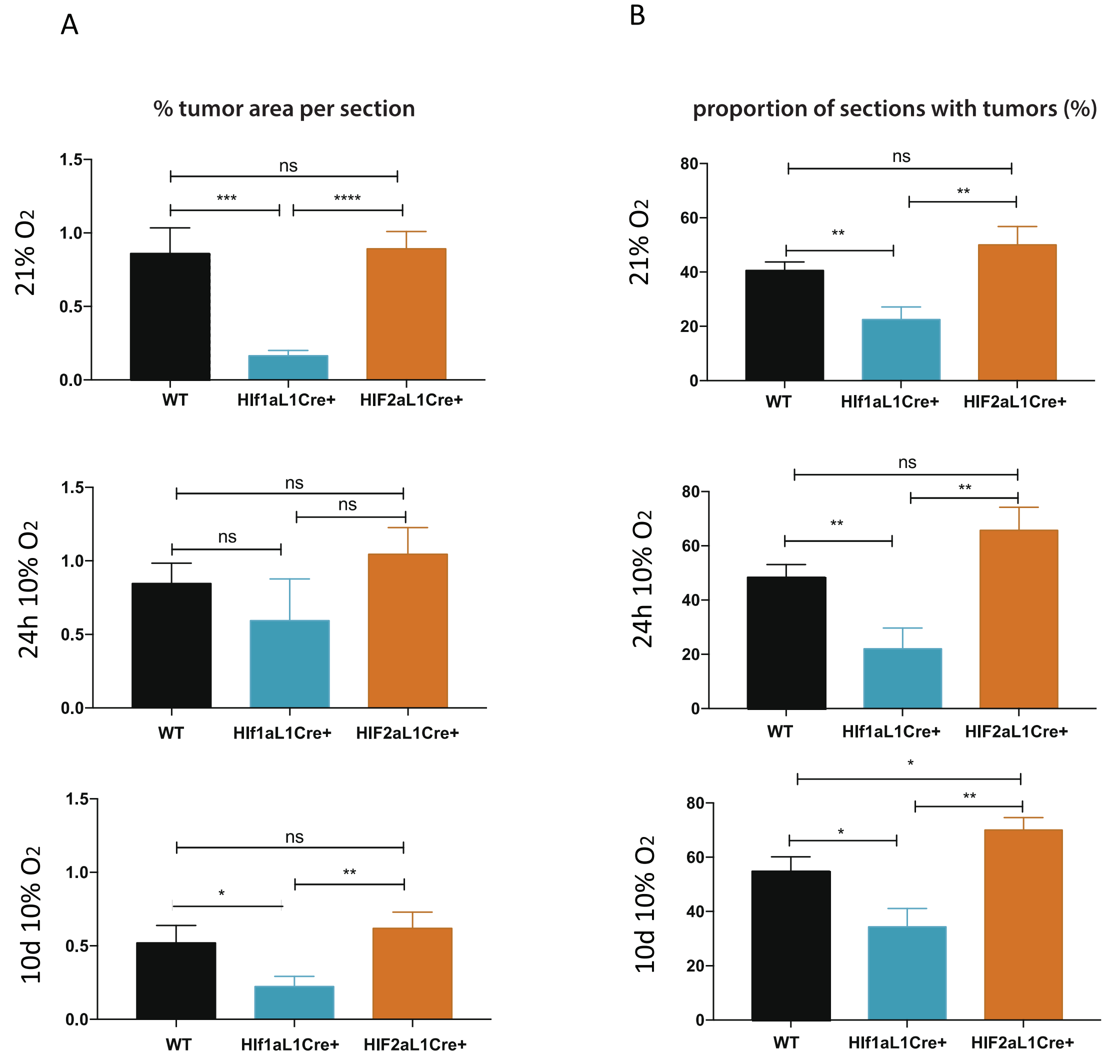

Endothelial HIF mediated metastatic incidence. (A) Lung tumours were quantified to assess the proportion of tumour area to total area of lung section in control (DF) and mutant animals (tumour area/total area x 100), following different hypoxia challenges; (B) The average proportio n of sections containing tumours (sections with tumours/total number of evenly-spaced lung sections x 100) in HIF-1αL1 or HIF-2αL1 mutants and their respective L1Cre-negative litter mate controls. Statistical significance was assessed by Student's *t*-test with Welch's correction: *p<0.05, ** p<0.01, *** p<0.001.

Supplementary Fig. 5

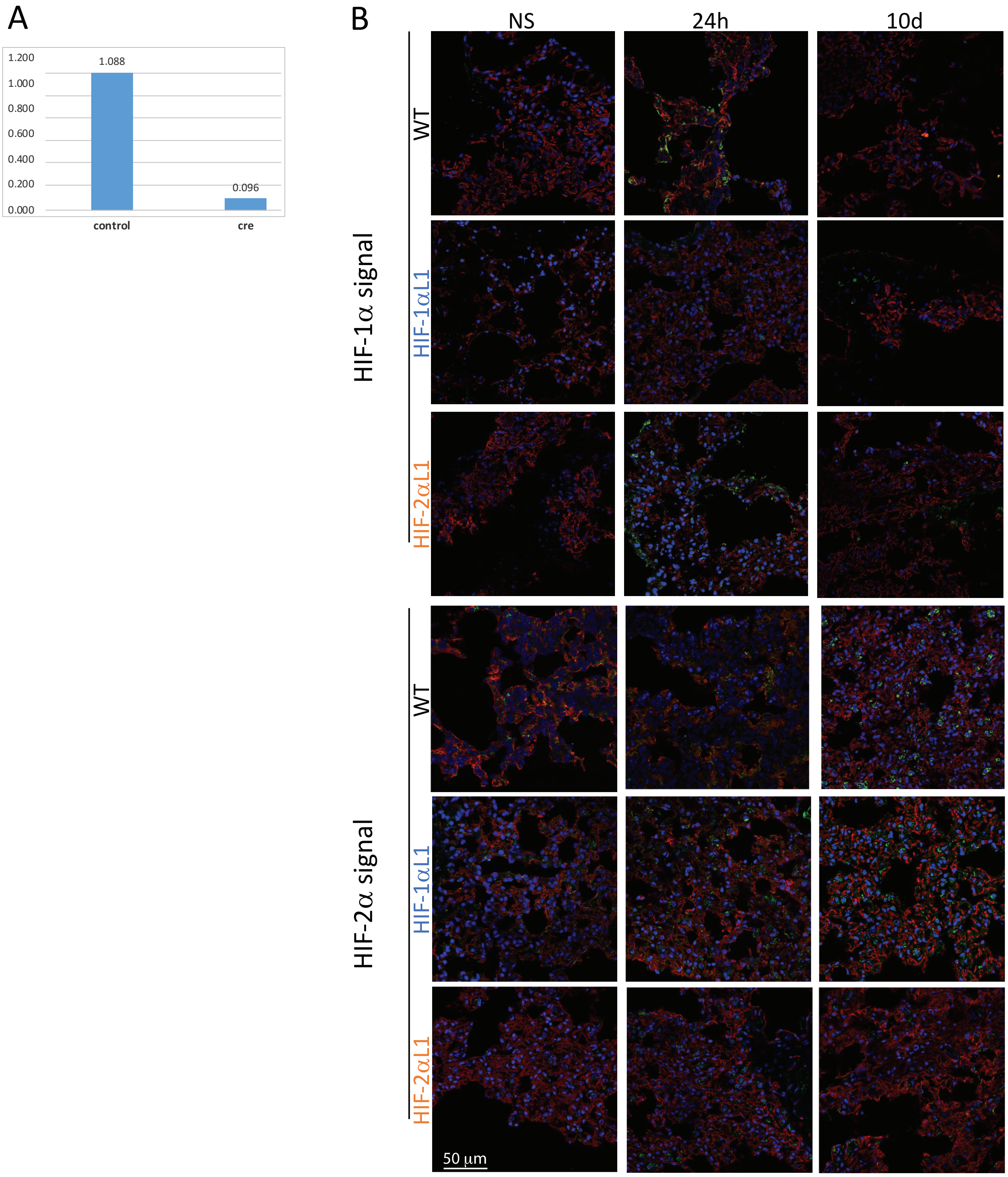

Effect of of Cre expression on HIF-α deletion. (A) Deletion of HIF-1α in primary EC carrying double-floxed HIF-1α allele and exposed to Cre-expressing Adenovirus is consistently above 90% (WT control cells were treated with adenovirus expressing β-galactosidase); (B) Representative IF images of HIF-α isoform signal in all treatments and all genotypes; Animals expressing lung endothelium specific L1 Cre show visibly less HIF signal for the respective isoform.

Supplementary Fig. 6

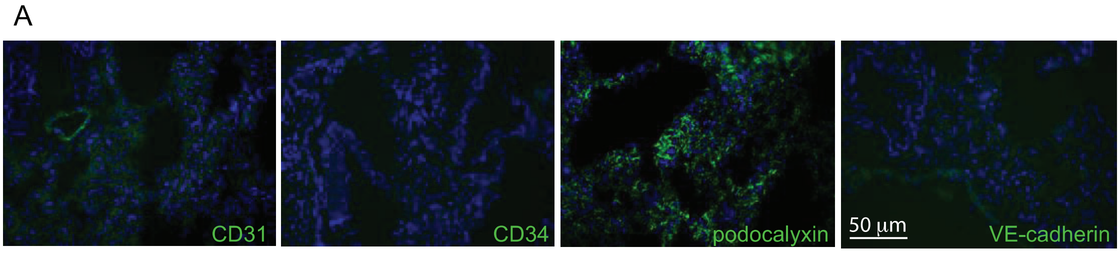

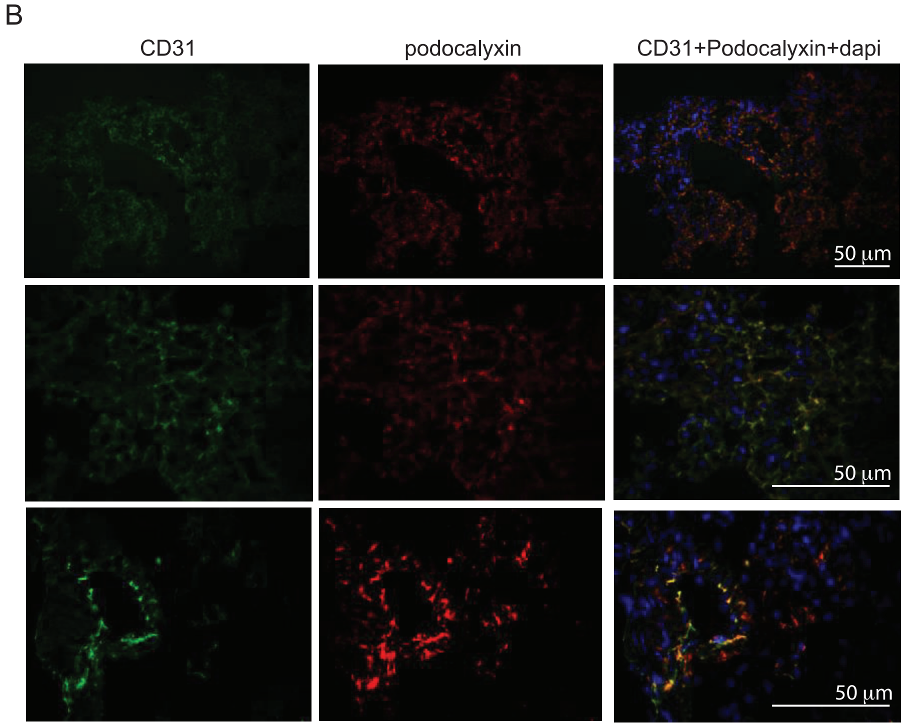

Validation and optimization of endothelial cell IF staining. (A) Staining and IF of EC in deflated and not perfused lung tissue, using multiple commonly used endothelial cell markers; (B) validation of specificity of Podocalyxin by co-staining and co-localization with CD31.

Supplementary Fig. 7

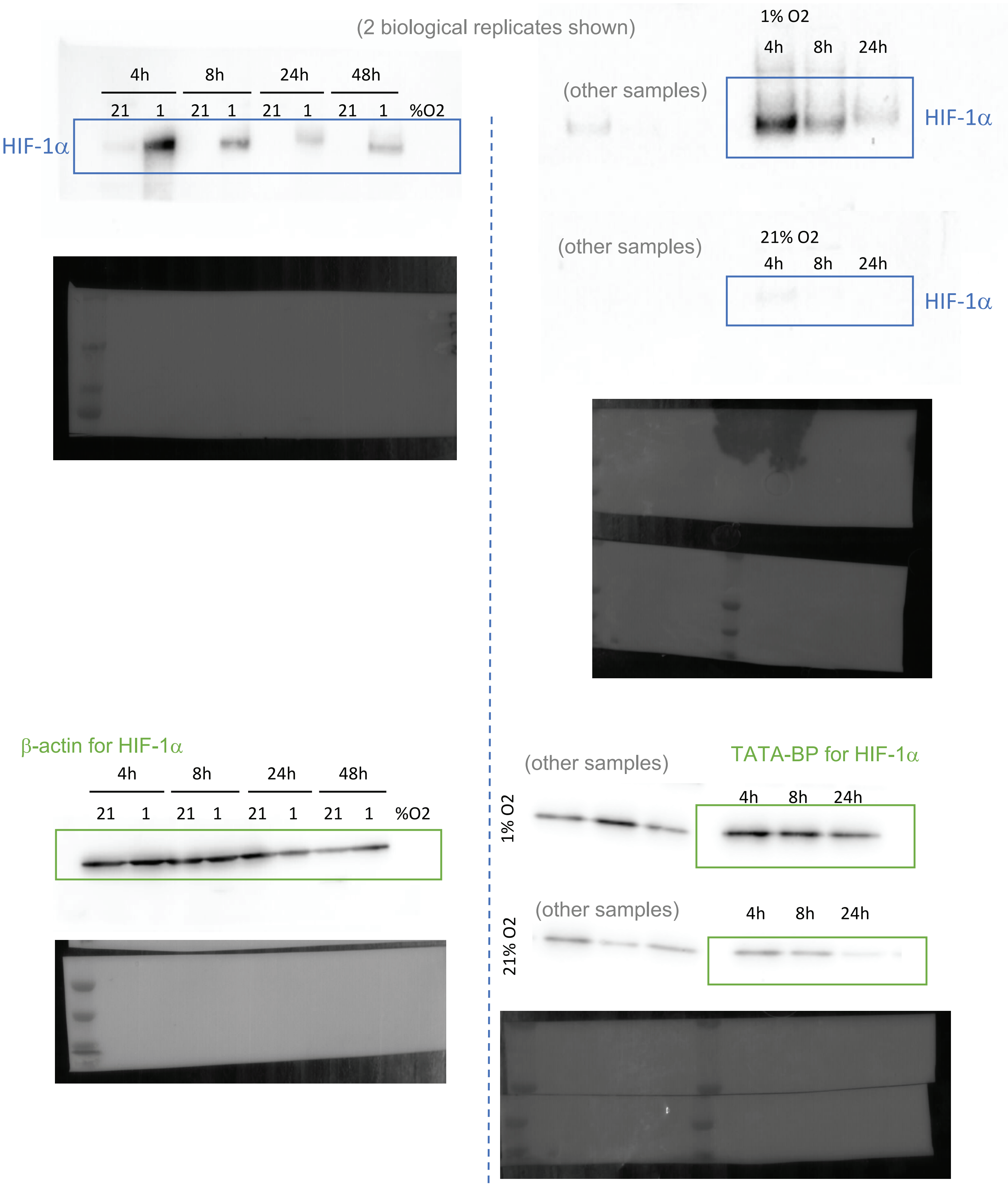

Original whole membrane images of WB using nuclear extracts of normoxic and hypoxic primary cells probed for HIF-1α, cropped and shown in main text. PVDF membranes were cut at 75 KDa (using BioRad Precision Plus dual collor ladder). Bottom halft was used to probe for β-actin or TATA-binding protein, and upper half for HIF-1α. Multiple membranes were occasionally scanned at once when target signal intensity was comparable. Sections framed in blue represent for HIF-1α and green β-actin control.

Supplementary Fig. 8

Biological replicate 1

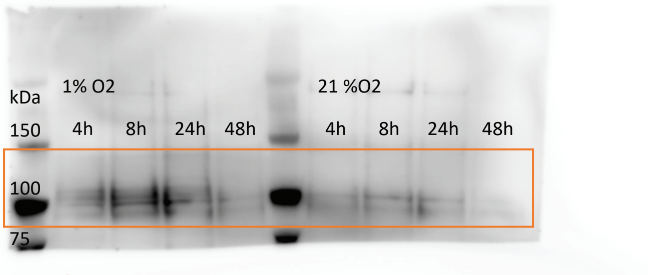

HIF-2a

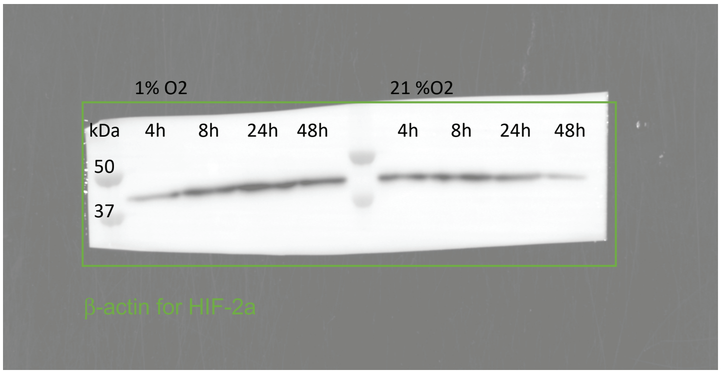

Biological replicate 2

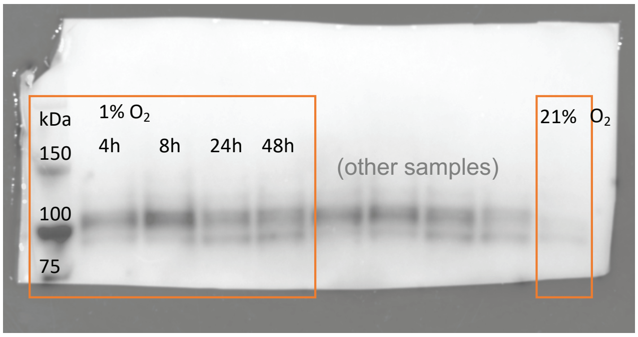

HIF-2a

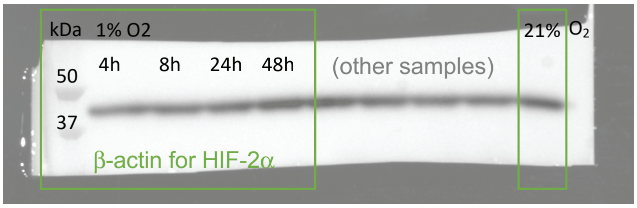

Whole scans of western blot of nuclear extracts of primary cells
probed for HIF-2α, cropped and shown in main text (biological replicate 2). PVDF membranes were cut at 75 KDa (using BioRad Precision Plus dual colour ladder). Bottom half was used to probe for β-actin
and upper half for HIF-2α. Multiple membranes were occasionally scanned at once when target signal intensity was comparable. Sections framed in
orange represent for HIF-2α and green β-actin control, in the whole scan images.

Supplementary Fig. 9

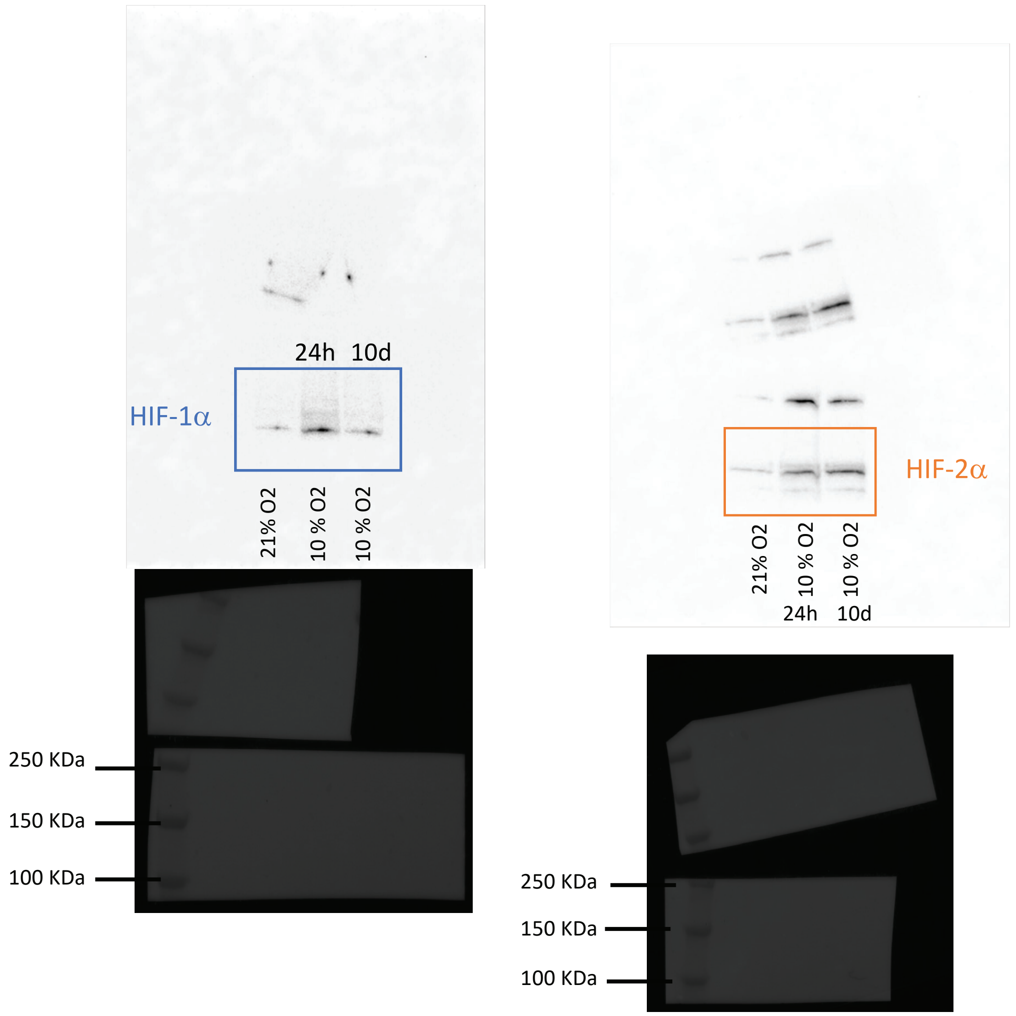

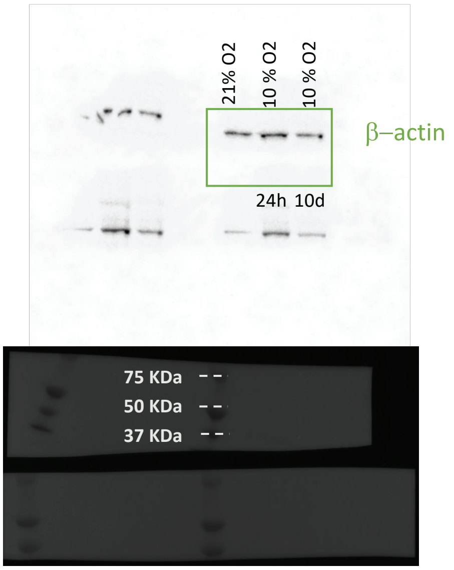

Scans of western blot membranes of whole lung protein probed for HIF-1α, HIF-2α and β-actin control, cropped and shown in the main text. PVDF membranes were cut at 75 KDa (using BioRad Precision Plus dual colour ladder). Samples were prepared in one large mix and same volume (corresponding to 15ug of protein) were loaded in multiple wells, to probe for multiple targets. The upper half of the membranes was used for targets running above 75 KDa, and the bottom half was used to probe for β-actin and other targets smaller than 75 KDa. Cropped sections used in main figure are framed in blue for HIF-1α, orange for HIF-2α and green for β-actin control.
Images of the membranes are provided. These were superimposed to the luminescence images to locate the targets in relation to the standards. The membrane used for HIF-2α probe was also used for iNOS (shown in Supplementary fig. 2D).

Extended Methods

Animal models: Deletion of *HIF-1*α or *HIF-2*α in lung EC was obtained by crossing female animals homozygous for the floxed alleles of *HIF-1*α[1] or *HIF-2*α[2] (double-floxed, DF) to *HIF-1*α^{df}*/L1Cre*$^{+}$ or *HIF-2*α^{df}*/L1Cre*$^{+}$ males[3]. Experimental cohorts including L1Cre^{+} were perfomed using Cre- littermate controls (DF animals with no deletion, physiologically wild-type, as previously shown[1,2]). We have previously shown that L1-driven Cre expression is confined to the pulmonary endothelium[4]. C57Bl/6 WT mice were purchased from the Charles River laboratories (UK).

In vivo Hypoxia treatments: Eight-week old male animals were exposed to 10% O_2 for either 24 h (acute exposure, optimised for preferential activation of HIF-1α in lung tissue) or 10 d (chronic exposure, optimised for preferential activation of HIF-2α in lung tissue), in chambers with controlled humidity and CO_2 (Biospherix). Control animals were maintained in normal atmosphere. Tissues were collected immediately after hypoxia exposure and either fast frozen for protein or RNA extraction, processed for flow cytometry or placed in OCT compound (Tissue-Tek, Cat # 4583) for histological analyses. Lung tissue hypoxia upon exposure to 10% O2 was confirmed by pimonidazole (Hypoxyprobe, HP1-1000 kit). , according to manufacturers instructions; briefly, pimonidazole was administered at 30 mg/Kg in sterile saline via tail vein injection, and allowed to penetrate tissues for 90 min. Animals were sacrificed by cervical dislocation and lung tissue immediately collected into OCT medium for cryosectioning. 20 μM sections were fixed in acetone for 8 min and then stained with MAB1 antibody (included, 1:50 in PBS, at 4ºC, O/N) and detected with anti-mouse AF647 conjugated antibody (Abcam, ref. ab150127, 1:100 in PBS, 2h at RT).

Metastasis Assay: A total of 5×10^5 LLC cells in 200 µl sterile PBS were injected into tail veins of animals pre-conditioned in hypoxia or normal air controls. Injections were performed inside the hypoxia chamber for the hypoxic pre-conditioned animals, and animals were

subsequently placed in room air until tissue collection. Lungs Treatments were set-up such that all animals were exactly the same age at the time of injection (hypoxia treatments started at day -10 for chronic treatments, day -1 for acute treatments, and at day 0 all animals received tumour cells from the same culture batch). Animals were transferred to normal air after tumour cell injection. Lung tissue was collected either 24h (to monitor immediate responses) or 14 days post-injection (to quantify metastatic take). Lung tissue was collected directly into OCT, immediately processed for flow cytometry, snap frozen in liquid N_2 (for protein and RNA extractions), or perfused with 10 mg/mL heparin in PBS, and fixed in 4% PFA for paraffin embedding and H&E staining. Lung tumors were counted in H&E-stained 10 µm evenly spaced sections across whole lungs (≥ 20 sections per animal). Tumor area was quantified using Image J software[5].

Pulmonary vascular permeability: 50 mg/Kg of Evans Blue (Sigma, Cat. # E2129) dye in PBS were injected into the tail vein of each animal, and bronchoalveolar lavage was collected 20 minutes later in 1 mL of PBS. Evans Blue concentration in the lavage, an indication of leaked dye from the lung microvascular network, was quantified against a standard curve by spectrophotometry at 540 nm.

Flow cytometry: Lung tissue was digested for 20 min in Collagenase D (Roche, 500 µg / ml) / DNAse I (Roche, 50 µg/ml) at 37ºC, and neutralized with EDTA. The digested tissue was strained through a 70 µm filter and the filtrate was treated for 2 min with ACK lysing buffer (Thermofisher). Cells were stained with fluorophore-conjugated antibodies for 20 minutes at 4ºC, and data acquired on Fortessa (BD Biosciences). The fluorophore-conjugated antibodies used were: CD11b (eBioscience, M1/70, Alexa Fluor 700), NK-1.1 (Biolegend, PK136, PE), CCR2 (R&D systems, 475301, APC), Ly-6G (Biolegend, 1A8, PerCP/Cy5.5), CD11c (BD Bioscience, HL3, FITC), CD45.2 (Biolegend, 104, PE/Cy7), Ly-6C (BD Bioscience, AL-21,APC-Cy7), CD16/CD32 (BD Bioscience,2.4G2), Zombie Yellow (viability dye, Biolegend). Data analysis and gating was performed on FlowJo (FlowJo, LLC).

Endothelial cells were identified as $CD45^{-}CD31^{+}$ gated directly from viable cell population (Figure 2A). Macrophages (Figure 3D) were initially identified as CD45+CD11b+ from the viable cell pool, and these markers generally identify perivascular and activated macrophages. A “true macrophage” population (Figure 5C) was further identified by sequential subtractive gating of non-macrophage cells: CD45+ viable cell population was gated to exclude NK1.1 and CD16 (Natural Killer cells) and subsequently CD11b+CD11c+ cells (Dendritic cells), followed by removal of the neutrophil population (Ly6G+), finally isolating a classic “true” macrophage population (modified from Rose et al. 2012[6]).

<u>Immunochemistry/Immunofluorescence</u>: Deparaffinised or frozen-fixed sections were blocked with Universal Protein Blocking Reagent (GeneTex), followed by an incubation with 0.1% Sudan Black[7,8] in 70% Ethanol (10 min, RT) and Mouse-on-Mouse Blocking Reagent (Vector) when appropriate, and according to the supplier’s instructions. Primary antibody incubations occurred O/N at 4ºC (HIF-1α (NB100-105)[9] or HIF2α (NB100-132)[10], Podocalyxin (R&D Systems AF1556)[11], iNOS (NB300-605), VE-cadherin (Novus Biologicals, AF1002)). Secondary antibodies conjugated with either Alexa Fluor 488 (Thermofisher Scientific) or Alexa Fluor 647 (Abcam) were used (1 hour at RT, in the dark). Slides were mounted with ProLong Diamond antifade with DAPI (P36962,ThermoFisher Scientific) and imaged the following day. For primary EC staining, cells were plated and cultured in gelatine-coated glass slides and fixed immediately after hypoxia treatments in cold 4% PFA in PBS, for 20 min at 4ºC. Fixed cells were permeabilized with 0.4% Triton-X in PBS for 5 min, blocked with Universal Protein Blocking Reagent (as above), and protein targets were detected by overnight/4ºC incubations with primary antibodies, and 1h room temperature incubation with fluoro-conjugated secondary antibodies. Slides were mounted with Vectashield hardset antifade medium with DAPI (Vector, H-1500). Images were collected in a Leica fluorescent microscope and analysed in Image J[5].

TUNEL Assay Protocol was performed using a slightly modified version of manufacturer's instructions (Thermofisher- Click-iT® TUNEL Alexa Fluor® 488 Imaging Assay, #C10245); Slides containing *frozen tissue* sections were fixed in pre-cooled acetone for 10 minutes; slides with primary *endothelial cells* were instead fixed with cold 4% PFA for 20 min at 4°C. Following washes, all samples were treated with 3% H_20_2 in MeOH for 10 min, sections were blocked and permeabilized with 0.4% Triton-X in PBS for 5 min, and lung tissue sections were further incubated with Sudan Black B (Sigma, Cat # 199664) for 10 min to reduce autofluorescence. Equilibration with TdT for 10 min at RT preceded a 1h incubation with TdT reaction cocktail (TdT reaction buffer, EdUTP, TdT*), at 37°C in a humidified chamber. Slides were washed with 3% BSA in PBS for 5 min. Signal was generated with Click-iT reaction buffer and additive, according to manufacturer's protocol. Secondary immunostaining, when performed, was done after this step. All slides were mounted in Vectashield anti-fade mounting medium with DAPI (Vector) and imaged the following day.

Microscopy and image analysis: Images were taken with Leica fluorescence microscope using a x20, x40 or x63 (oil immersion) objective. Gain and offset were set to negative controls, and used to standardize image acquisition for each experiment (tissue: three images per section, a ≥ 8 randomly chosen sections from each animal; cells: ≥ 4 images per slide chamber, three slides per treatment). Quantification of fluorescence was done using ImageJ. After setting the threshold to a duplicate image, stained areas are identified and selected. "Dark background" is selected for fluorescence. "Analyze-Analyze particles" was used for measurement of various parameters; background values were acquired by selecting three random areas in gray-scale image using the circle tool and "Analyze-Measure" function. Quantification in tissue samples was performed as follows:

$$Mean\ cell\ fluorescence = \frac{Integrated\ density}{Area}$$

The mean fluorescence for each image was calculated:

$$Mean\ fluorescence\ for\ background = \frac{Integrated\ density}{Area}$$

To correct for uneven fluorescence the following formula was used:

$$Correction\ factor = Mean\ cell\ fluorescence - Mean\ fluorescence\ for\ background$$

The number of TUNEL^{+} cells was identified by DAPI co-staining.

Confocal Imaging and co-localization: Images were acquired in a Leica SP5 confocal microscope using x20, x40 or x63 (Oil Immersion) objective. Images were acquired through LAS (Leica) and analysed using ImageJ [5]. Pearson's correlation was calculated to assess co-localization through the Coloc 2 plugin.

qPCR: For quantification of steady-state mRNA levels, total RNA was extracted from fast-frozen lung tissue or cells, using RNeasy columns (QIAGEN) with DNaseI in-column treatment (QIAGEN). cDNA was synthesized using Superscript III (Invitrogen) according to the manufacturer's instructions. Relative abundance of transcripts of interest was assessed by quantitative real-time PCR and normalised to β-*ACTIN* transcript levels. Primer pair and primer/probe sets for iNOS, VEGF, Arginase II and β-actin were reported elsewhere [12], primers for BNIP3 were designed for amplification with SYBR-green reagents (Roche) and are BNIP3 Fwd:GACGAAGTAGCTCCAAGAGTTCTCA, and BNIP3 Rev: CTATTTCAGCTCTGTTGGTATCTTGTG; VE-Cadherin primer set was obtained from Qiagen (Cat. No. QT000110467). Data are presented as average fold-change (ratio) ± SD (or SEM, as stated in each figure), between each sample and the respective control. Plots and statistical analysed was performed using Prism 7 software, and significance assessed by Student's *t*-tests with Welch's correction or ANOVA.

Cell culture and hypoxia treatments: Primary EC were isolated and cultured from lungs of *HIF-1*α^{df}, *HIF-2*α^{df}, *iNOS*^{df} male animals between 6 and 8 weeks of age, as previously described [12]. Cre recombinase-mediated gene deletion was performed *ex-vivo* by overnight

incubation with Cre Recombinase Gesicles (Clontech, cat. No. 631449), or with Adenovirus Cre, in which case control double-floxed cells were infected with β-galactosidase expressing Adenovirus; Cells were allowed to recover for 24h post-gene deletion before experiments were performed. Hypoxia (1% O_2) treatments were performed in cultures no later than Passage 3.

<u>*Ex-vivo* endothelial monolayer permeability</u>: Primary cells were plated onto Fluroblok 8μm inserts (light-blocking PET, Corning), placed in 24-well companion plates (Falcon, Cat # 353504), in Boyden chamber-like set up. Cells were maintained with 200 μL media on the upper compartment. When confluent, the basal media inside the insert was replaced by 150 μL media containing 1mg/mL FITC Dextran 70 KDa, and 250 μL FITC Dextran-free media was placed in the lower chamber, all pre-equilibrated at 1% O_2. Plates with inserts were placed inside plate reader at 1% O_2, and fluorescence was measured in the lower compartment, as an indication of FITC leaked through the endothelial monolayer to the lower chamber. Readings were taken every 5 minutes for 4 hours.

<u>Western blotting</u>: Equal amounts of protein (15 μg) were obtained from either cells or whole lung tissue lysed in RIPA buffer, resolved in 3–8% acrylamide Tris-Acetate gels (Life Sciences, EA0375BOX) and transferred to PVDF membrane using BioRad Transblot® Turbo™ Transfer System. Primary antibodies were used at 1:1,000 (iNOS, sc-651; HIF-1α, NB-100-049) or 1:500 dilutions (HIF-2α, R&D AF2997). Membranes were cut at 75KDa band and targets were probed on the upper half of the membrane, whereas β-actin (Sigma, A1978, running at ~50KDa), used as normalization control, was probed on the bottom half of the membrane. Membranes were not re-probed for multiple antibodies. Protein signals were detected following secondary incubation with HRP-conjugated antibodies for 1h at room temperature, and ECL Plus chemiluminescence detection kit (Amersham, Cat. # RPN2232), according to manufacturer's protocol. Image capture and quantification were performed

using FusionFX (Vilber), quantification were performed using FusionFX (Vilber) using default exposure for optimal signal detection. Statistical analysis was done using unpaired Student's *t*-test using Welch’s correction, with GraphPad Prism software. Data are expressed as mean ± SD or SEM, as stated for each dataset.

References:

1 Ryan, H. E., Lo, J. & Johnson, R. S. HIF-1 alpha is required for solid tumor formation and embryonic vascularization. *EMBO J.* **17**, 3005-3015, doi:10.1093/emboj/17.11.3005 (1998).

2 Gruber, M. *et al.* Acute postnatal ablation of Hif-2alpha results in anemia. *Proc. Natl. Acad. Sci. U. S. A.* **104**, 2301-2306, doi:10.1073/pnas.0608382104 (2007).

3 Park, S. O. *et al.* ALK5- and TGFBR2-independent role of ALK1 in the pathogenesis of hereditary hemorrhagic telangiectasia type 2. *Blood* **111**, 633-642, doi:10.1182/blood-2007-08-107359 (2008).

4 Cowburn, A. S. *et al.* HIF2alpha-arginase axis is essential for the development of pulmonary hypertension. *Proc. Natl. Acad. Sci. U. S. A.* **113**, 8801-8806, doi:10.1073/pnas.1602978113 (2016).

5 Schindelin, J. *et al.* Fiji: an open-source platform for biological-image analysis. *Nature methods* **9**, 676-682, doi:10.1038/nmeth.2019 (2012).

6 Rose, S., Misharin, A. & Perlman, H. A novel Ly6C/Ly6G-based strategy to analyze the mouse splenic myeloid compartment. *Cytometry. Part A : the journal of the International Society for Analytical Cytology* **81**, 343-350, doi:10.1002/cyto.a.22012 (2012).

7 Jenvey, C. J. & Stabel, J. R. Autofluorescence and Nonspecific Immunofluorescent Labeling in Frozen Bovine Intestinal Tissue Sections: Solutions for Multicolor Immunofluorescence Experiments. *J. Histochem. Cytochem.* **65**, 531-541, doi:10.1369/0022155417724425 (2017).

8 Sun, Y. *et al.* Sudan black B reduces autofluorescence in murine renal tissue. *Arch. Pathol. Lab. Med.* **135**, 1335-1342, doi:10.5858/arpa.2010-0549-OA (2011).

9 McLaren, A. T. *et al.* Increased expression of HIF-1alpha, nNOS, and VEGF in the cerebral cortex of anemic rats. *Am. J. Physiol. Regul. Integr. Comp. Physiol.* **292**, R403-414, doi:10.1152/ajpregu.00403.2006 (2007).

10 Schokrpur, S. *et al.* CRISPR-Mediated VHL Knockout Generates an Improved Model for Metastatic Renal Cell Carcinoma. *Sci. Rep.* **6**, 29032, doi:10.1038/srep29032 (2016).

11 Cunha, S. I. *et al.* Endothelial ALK1 Is a Therapeutic Target to Block Metastatic Dissemination of Breast Cancer. *Cancer Res.* **75**, 2445-2456, doi:10.1158/0008-5472.Can-14-3706 (2015).

12 Branco-Price, C. *et al.* Endothelial cell HIF-1alpha and HIF-2alpha differentially regulate metastatic success. *Cancer Cell* **21**, 52-65, doi:10.1016/j.ccr.2011.11.017 (2012).
